# Mononucleotide repeats are asymmetrically distributed in fungal genes

**DOI:** 10.1186/1471-2164-9-596

**Published:** 2008-12-11

**Authors:** Mark WJ van Passel, Leo H de Graaff

**Affiliations:** 1 Laboratory of Microbiology, Wageningen University, Dreijenplein 10, 6703 HB, Wageningen, the Netherlands

## Abstract

**Background:**

Systematic analyses of sequence features have resulted in a better characterisation of the organisation of the genome. A previous study in prokaryotes on the distribution of sequence repeats, which are notoriously variable and can disrupt the reading frame in genes, showed that these motifs are skewed towards gene termini, specifically the 5' end of genes. For eukaryotes no such intragenic analysis has been performed, though this could indicate the pervasiveness of this distribution bias, thereby helping to expose the selective pressures causing it.

**Results:**

In fungal gene repertoires we find a similar 5' bias of intragenic mononucleotide repeats, most notably for *Candida *spp., whereas e.g. *Coccidioides *spp. display no such bias. With increasing repeat length, ever larger discrepancies are observed in genome repertoire fractions containing such repeats, with up to an 80-fold difference in gene fractions at repeat lengths of 10 bp and longer. This species-specific difference in gene fractions containing large repeats could be attributed to variations in intragenic repeat tolerance. Furthermore, long transcripts experience an even more prominent bias towards the gene termini, with possibly a more adaptive role for repeat-containing short transcripts.

**Conclusion:**

Mononucleotide repeats are intragenically biased in numerous fungal genomes, similar to earlier studies on prokaryotes, indicative of a similar selective pressure in gene organization.

## Background

Genetic patterns could not be studied comprehensively until whole genome sequences became available. The first such genome-wide sequence analysis focused on both the abundance and distribution of competence-associated sequence motifs in the prokaryote *Haemophilus influenzae *[1]. Since then, as more genome sequences became available, numerous other genetic features were studied in prokaryotes [2-4] and eukaryotes [5,6]. Many such genome-wide analyses concentrated on the genomic distribution of simple sequence repeats (SSRs, also known as microsatellites), stretches of mono- and oligonucleotide repeats [7-10].

SSRs generally occur more frequently in non-coding regions of the genome. One of the reasons for this avoidance in the protein coding regions is that many SSRs predispose for disruptive frameshifts via strand-slippage during replication, transcription or translation [8,11]. Recently Ackermann and Chao postulated that selection for sequence stability in coding regions has been a pervasive force in the distribution biases of mononucleotide repeats (MNRs) in both prokaryotic and eukaryotic genome sequences [12]. More recently, we discovered that MNRs also display a biased distribution within coding sequences: in a wide range of both bacterial and archaeal genomes, mononucleotide repeats were predominantly biased towards the 5' end of genes [13], presumably to prevent or reduce the expression of toxic or costly frameshifted proteins. However, for eukaryotes no such intragenic distribution analysis has been carried out.

The Fungi represent a kingdom within the eukaryotic domain with many fully sequenced representatives. These can vary in genome size (less than 3 Mbp for *Encephalitozoon cuniculi*, up to an estimated 82 Mbp for *Puccinia graminis*), cellular organization (single cells or multicellular organisms) as well as life-style (saprophytic or pathogenic) [14,15]. With respect to SSRs, a previous analysis in sequenced fungal genomes described different patterns of their occurrence [9], but did not study their intragenic distribution.

Here we analyze genome-wide sets of predicted coding sequences from fully sequenced fungal genomes, and assess the intragenic distribution patterns of mononucleotide repeats. MNRs are more commonly present than the more complex repeats, which makes their interspecific distribution comparisons possible. This could illuminate the similarities and differences between the distributions of disruptive repeats in prokaryotes and eukaryotes, and help identify the selective forces that brought about these biases.

## Methods

Transcript data of the coding regions (excluding introns) from 47 fungal genomes were obtained from the Broad Institute . The datasets, their size and the description of the strains are given in Table 1. Truncated genes, transcripts with annotated internal stop codons and genes that are not multiples of three in length were excluded from the analyses, which basically rely on a genome-wide quantitative assessment of MNRs in the five quintiles of the annotated protein-coding sequences. Sequence motif analyses and codon usage profiles were carried out using in-house perl scripts, which are available upon request.

**Table 1:** List of the 47 analyzed species, the number of coding sequences and their scientific, industrial or biomedical merit.

**Name**	CDS*	**Description of strain relevance**
*Aspergillus clavatus*	9110	Animal pathogen
*Aspergillus flavus*	12434	Phytopathogen
*Aspergillus fumigatus*	9884	Opportunistic human pathogen
*Aspergillus nidulans*	10474	Model organism
*Aspergillus niger*	6329	Industrial strain
*Aspergillus oryzae*	12063	Industrial strain
*Aspergillus terreus*	10400	Opportunistic human pathogen/industrial strain
*Batrachochytrium dendrobatidis*	8779	Amphibian pathogen
*Botrytis cinerea*	15512	Phytopathogenic fungus
*Candida albicans sc5314*	5916	Opportunistic human pathogen
*Candida albicans wo1*	5851	Opportunistic human pathogen
*Candida guilliermondii*	5897	Haploid relative of Candida albicans
*Candida lusitaniae*	5891	Opportunistic human pathogen
*Candida parapsilosis*	5687	Opportunistic human pathogen
*Candida tropicalis*	6216	Opportunistic human pathogen
*Chaetomium globosum*	10987	Important decomposers of biomass
*Coccidioides immitis h538.4*	10480	Human pathogen
*Coccidioides immitis rmscc 2394*	10368	Human pathogen
*Coccidioides immitis rmscc 3703*	10379	Human pathogen
*Coccidioides immitis rs*	10609	Human pathogen
*Coccidioides posadasii rmscc 3488*	9932	Human pathogen
*Coccidioides posadasii str. silveira*	10070	Human pathogen
*Coprinus cinereus*	13523	Model organism for multicellularity
*Cryptococcus neoformans h99*	7077	Human pathogen
*Debaryomyces hansenii*	6101	Cryo- and halotolerant marine yeast
*Fusarium graminearum*	13220	Representative of an important family of phytopathogens
*Fusarium oxysporum f. sp. lycopersici*	17202	Phytopathogen and model organism for evolutionary research
*Fusarium verticillioides*	14002	Phytopathogen and model organism for evolutionary research
*Histoplasma capsulatum nam1*	9164	Human pathogen
*Lodderomyces elongisporus*	5739	Closest sexual relative to *Candida albicans*
*Magnaporthe grisea*	12564	Phytopathogen
*Neosartorya fischeri*	10383	Opportunistic human pathogen and involved in food spoilage
*Neurospora crassa*	9795	Model organism
*Paracoccidioides brasiliensis pb03*	9235	Human pathogen
*Puccinia graminis f. sp. tritici*	20462	Phytopathogen
*Pyrenophora tritici-repentis*	12092	Phytopathogen
*Rhizopus oryzae*	17074	Representative agent of mucormycosis
*Saccharomyces cerevisiae rm11-1a*	5272	Model organism
*Schizosaccharomyces japonicus yfs275*	5122	Model organism for comparative genomics study
*Schizosaccharomyces octosporus yfs286*	4906	Model organism for comparative genomics study
*Schizosaccharomyces pombe 972h*	4991	Model organism
*Sclerotinia sclerotiorum*	13704	Broad host range phytopathogen
*Stagonospora nodorum*	15949	Phytopathogen
*Uncinocarpus reesii*	7777	Closest known relative to the pathogenic Coccidioides
*Ustilago maydis*	6517	Phytopathogen
*Verticillium albo-atrum vams.102*	10098	Phytopathogen
*Verticillium dahliae vdls.17*	10453	Phytopathogen

*) All transcripts, except those with annotated internal stop codons, annotated as missing ends or transcripts that are not multiples of three in length.

Fungal transcripts were compared to the KOG database [16] according to the nearest-neighbor method (using raw scores) [17] in order to distinguish between transcripts with a well-defined ortholog and transcripts that lacked an ortholog in the database.

## Results

### Intragenic distribution biases of mononucleotide repeats in fungal coding sequences

**Figure 1:**
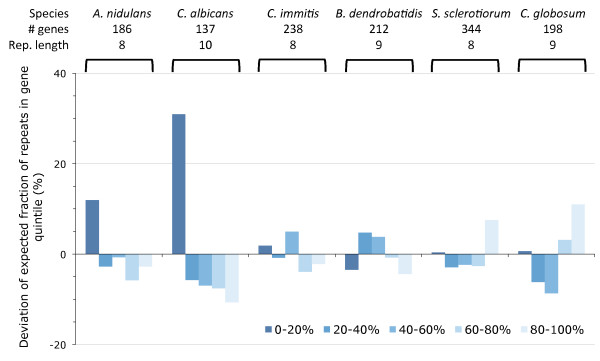
**Six examples of repeat distribution profiles in the gene quintiles of fungal gene repertoires.** Represented are the deviations of the expected value per quintile (i.e., 20%). # genes signifies the number of genes with mononucleotide repeats (per genome), and Rep. length signifies the length of the MNR in basepairs.

For each genome we tested the intragenic distribution profiles of the longest MNRs in the five quintiles of all predicted protein coding genes, with a minimal occurrence of 100 repeats of that length in the genomic transcript data (see Figure 1 for 6 representative cases, and Additional File 1 for all repeat distributions). The gene repertoires of most strains (25/47 genomes) show a non-proportional distribution of MNRs over the five quintiles (Chi-square, 4 degrees of freedom, p < 0.05, Additional File 1). The strongest bias is observed in *Candida parapsilosis*, where83% (104/125 repeats) of all repeats of 10 bp or longer are in the first quintile of the genes. In several cases, the majority of repeats are in the last quintile (80–100%) of the genomic gene set (e.g., *Botrytis cinerea *and *Chaetomium globosum*).

### Genes with mononucleotide repeats of 15 bp or longer

**Figure 2:**
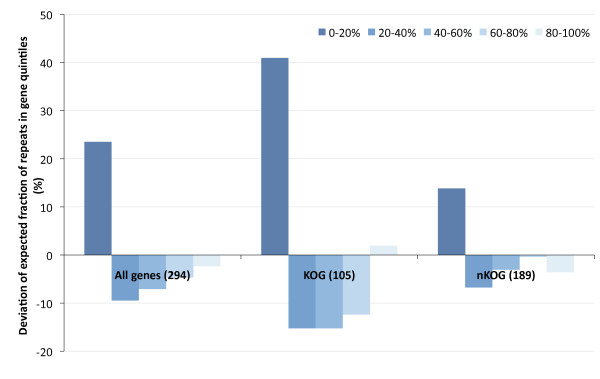
**The distribution of very long repeats (>15 bp mononucleotide repeats, 298 repeats, 294 genes) in the quintiles of the predicted coding regions from all tested fungal genomes.** Represented are the deviations of the expected value per quintile (i.e., 20%).

The 294 fungal transcripts with repeats of 15 residues or longer from all genomes combined are given in Additional File 2. Again, when analyzing the intragenic distribution of the 298 repeats in these 294 fungal genes, a strong bias towards the first quintile of the genes is observed, both in transcripts with well-described functional orthologs (KOGs), but also in transcripts without orthologs (nKOGs) in the KOG database (Figure 2). As for the extreme cases, *Aspergillus fumigatus*, *Aspergillus niger*, *Aspergillus terreus*, *Candida lusitaniae*, *Histoplasma capsulatum*, *Paracoccidioides brasiliensis and Rhizopus oryzae *each contain genes with mononucleotide repeats over 30 nucleotides long. The gene with the longest repeat is encountered in *A. terreus *(transcript ATET_00185), which contains a stretch of 68 consecutive adenine residues, encoding 22 consecutive lysines. Among the intragenic repeats of over 15 nucleotides in length, guanine and cytosine tracts are relatively rare with only 80 out of 298 repeats, similar as to what was found previously [18]. Still, some genomes harbour transcripts with repeats that consist solely of long guanine or cytosine tracts; *Chaetomium globosum *(6 genes), *Coprinus cinereus *(4 genes), *Neurospora crassa *(4 genes) and *Sclerotinia sclerotiorum *(5 genes). Other genomes have repeat-containing genes with only adenine or thymine tracts of over 15 residues in length: *Candida albicans *wo1 (11 genes), *Candida lusitaniae *(13 genes), *C. tropicalis *(41 genes), *Coccidioides immitis *rs (8 genes) and *R. oryzae *(11 genes).

### Trinucleotide repeats in *Neurospora crassa *coding regions

**Figure 3:**
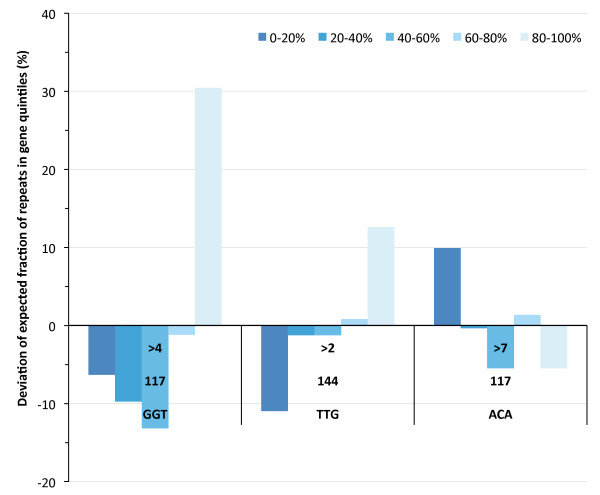
**Distribution of trinucleotide repeats GGT, TTG and ACA in the quintiles of the protein coding genes from *Neurospora crassa*.** Represented are the deviations of the expected value (i.e., 20%).

Previous analyses have shown that fungal genomes also harbour oligonucleotide repeats, which on occasion have been associated with particular processes [19]. These repeats are mostly typical for the individual species, and though abundant, the total repeat-specific counts are still often less than 100 in the coding regions of a genome. However, in *N. crassa*, numerous trinucleotide repeats have been identified [20], of which only a few are encountered in large numbers in the coding regions: (GGT)_n _(117 repeats of n > 4), (TTG)_n _(144 repeats of n > 2) and (ACA)_n _(117 repeats of n > 7). Changes in the copy number of trinucleotides in a gene do not cause a shift in the reading frame, and therefore we hypothesized that these repeats need not be intragenically biased. Nevertheless, we observed that all three trinucleotide repeats occur more frequently at the gene termini of coding regions, with over half of all GGT repeats in the last quintile of the coding regions (Figure 3). This suggests that the bias of repeats may not be caused solely by their risk for frameshifts.

### Intragenic repeat resistance as indicated by genome repertoire analyses and consecutive homogenous codon usage profiles

**Figure 4:**
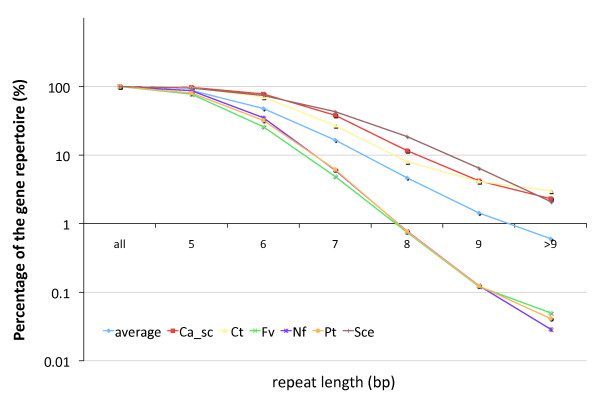
**Percentages of the gene repertoires that contain homopolymeric tracts in fungal species: only the three species with the highest (*C. albicans *sc5314, *C. tropicalis *and *S. cerevisiae*) and three lowest (*F. verticillioides*, *N. fischeri *and *P. tritici-repentis*) genome fraction that contain homopolymeric tracts of said length (x-axis) are depicted, as well as the average of all 47 strains.** Note the logarithmic scale on the y-axis. The data for all 47 strains is available in Additional File 3.

The fractions of genes per genome that contain a mononucleotide repeat decreases rapidly with increasing repeat lengths in all tested genomes (Figure 4). Nevertheless, a substantial difference may exist between the tolerances of the species to disruptive intragenic mononucleotide repeats. Short repeats (5 bp) are encountered in most (72–97%) of the protein-coding gene repertoires of all 47 tested species, and are never intragenically biased. However, with increasing repeat lengths, ever larger discrepancies arise between the percentages of the different genomic gene repertoires that contain repeats of such lengths (Figure 4, Additional File 3). *C. tropicalis *has an 80× larger gene fraction containing repeats of 10 residues or longer than *Neosartorya fischeri *(~3% and ~0.03%, respectively), although *N. fischeri *contains almost twice as many genes. This higher gene fraction with repeats suggests that *C. tropicalis *enjoys a much higher tolerance for disruptive intragenic repeats.

Comparing the expected versus observed frequencies of neighbouring lysine or phenylalanine codon, we find that *N. fischeri *avoids flanking homogeneous codons (*i.e*., AAA-AAA-AAA for lysine, and TTT-TTT-TTT for phenylalanine) to a much higher extent than *C. tropicalis*, corroborating the higher repeat tolerance of the latter species (data not shown).

### Intrageneric comparisons of intragenic mononucleotide repeats

The dataset contains the gene repertoires from different species and strains from the same genus, which allows us to test for intrageneric heterogeneity of repeat distribution biases. Three genera are tested, Aspergillus, Candida and Coccidioides. Firstly, both Aspergillus and Candida species show a 5' bias for intragenic repeat distribution patterns, which are relatively mild in Aspergillus, and strong in Candida. The sole outlier in the Aspergilli is *Aspergillus flavus*, which contrary to its relatives shows no (strong) 5' bias. In Candida, there is a very strong bias of repeats to the 5' end, except for the species *Debaryomyces hansenii*, which shows a 3' bias. The two species with the lowest observed 5' bias are *Candida guilliermondii *and *C. lusitaniae*, which are closely related species, and both branch off with *Debaromyces hansenii *in one of the so-called CTG-subclades [21]. Finally, none of the two tested Coccidioides species (eight strains) show a distribution bias in mononucleotide repeats.

### Authenticity of the intragenic repeat bias

**Figure 5:**
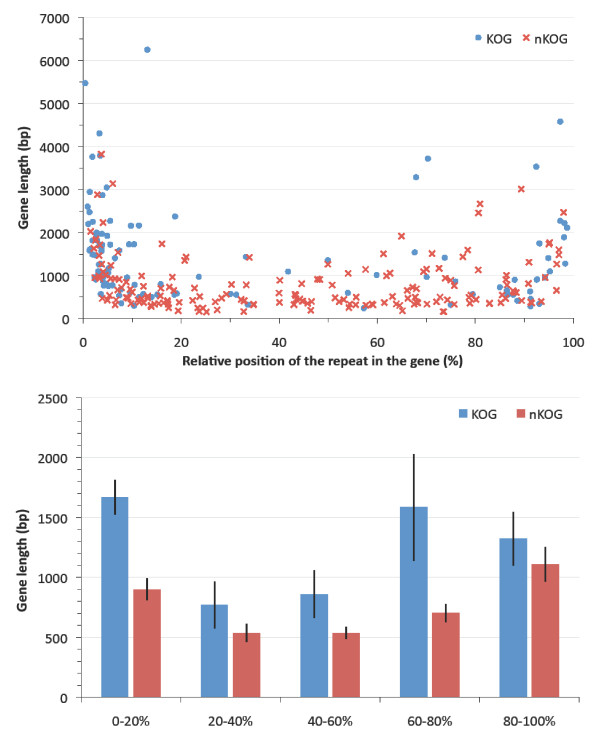
**A) Distribution of the relative position of long repeats (15 residues or longer) in a gene vs. the gene length.** Genes with a functional KOG annotation (KOG) and those without such an annotation (nKOG) are depicted with blue circles and red crosses, respectively. B) The average lengths of the transcripts (and the standard error of the means) are depicted for genes with a KOG annotation (blue) and genes without a KOG annotation (red), with respect to the position of the repeat in that transcript.

Of the 294 fungal transcripts that contain repeats of 15 bp or longer, 36% (105/294) could be assigned to a eukaryotic cluster of orthologous genes (KOG, Additional File 2, [16]). In this set of 105 orthologs to *bona fide *protein-coding genes, we observe a significant distribution bias of the repeats towards the 5' end (61% of all repeats are present in the first quintile of these 105 genes, p < 0.05, Figure 5A). Moreover, we discern that the genes with repeats at the gene termini are on average much longer than genes with the repeat in the middle of the gene (Figure 5B). A similar trend was observed for the fungal genes that could not be assigned to a KOG (termed nKOG), i.e., 34% of all repeats are in the first gene quintile, and transcripts with repeats at the gene termini are longer than transcripts with a repeat in the middle of the gene (Figure 5A and 5B). Interestingly, a large proportion of the 105 genes with long repeats that also have homologs in the KOG database are found in the pathogenic species *C. tropicalis *and *C. parapsilosis *(48 genes, 47 (98%) of which have the long repeat in the first gene quintile).

## Discussion and conclusion

Analyses of prokaryotic gene repertoires revealed a persistent bias of disruptive sequence repeats towards gene termini, potentially to curtail the metabolic costs or toxicity associated with transcribing and (or) translating non-functional genes [13]. In order to explore this phenomenon for the eukaryotic domain, we investigated the genome repertoires from 47 sequenced fungi, and discovered that this pattern is also evident in the fungal kingdom.

Still, large discrepancies exists between the intragenic distribution bias of MNRs of the different genomes: *Coccidioides *spp. show no intragenic preference for sequence repeats, whereas the *Candida *spp. demonstrate a very strong 5' end bias, with even up to 83% of all intragenic repeats in *C. parapsilosis *in the first gene quintile. Some genomes display a strong 3' end bias for intragenic repeats, such as *Botrytis cinerea*, which has 36% of its mononucleotide repeats in the last gene quintile. A bias of these disruptive repeats to either gene terminus agrees with a selection pressure to remove potential toxic side products, or alternatively to reduce metabolic costs, similar to what was found by Akashi and Gojobori in a comparison of 'expensive' and 'cheap' amino acid usage in highly expressed genes [22].

The species-specific gene fractions that contain long repeats could be a proxy for the species' tolerance for disruptive repeats. These differences are substantial, as the gene fraction of *C. tropicalis *with repeats of ten nucleotides or longer is 80 times higher than the gene fraction in *N. fischeri*. The adjacent homogeneous codon usage in these species also shows a higher avoidance of contiguous AAA and TTT codons in the latter species. A mechanistic explanation for a higher repeat-tolerance is still unknown, but could be studied by a more detailed functional characterisation of the genes that contain these long repeats.

When analysing the most abundant intragenic trinucleotide repeats in *N. crassa*, we find a strong bias of these repeats to the gene termini, even though differences in trinucleotide repeats do not cause a shift in the reading frame. This suggests that a selection pressure to remove potential toxic side products, or alternatively to reduce metabolic costs may not be the only explanation for the bias of repeats to gene termini. Previous analyses on amino acid repeats in *Drosophila *spp. also showed an avoidance of these repeats in the middle of genes [23]. Numerous trinucleotide repeat disorders are known to cause disease in humans [24,25], and studies into the intragenic repeat location biases could help determine mechanistic aspects of repeat expansions and contractions.

Sequence repeats have been thought to convey adaptive benefits due to their potential to facilitate rapid changes in the coding content [26], while one study also suggested that repeats are preferentially located towards recombination hot spots [27]. We find that larger genes have a more prominent bias of repeats at their gene termini. This could mean that many of the smaller transcripts with their mononucleotide repeat in the middle are misannotations, or gene remnants after the erosion of repeat-containing genes. Alternatively, these small repeat containing genes could represent foci of genetic novelty.

This focus on intragenic distribution biases is new [13,28], and could be expanded to other features previously not analysed as such, like methylation patterns, or targets for transcriptional or translational regulation. This can help resolve the origin and organization of new genetic features, as well as the evolutionary forces governing them.

## Authors' contributions

MWJvP conceived the project, carried out the analyses, interpreted the results and wrote the paper. LHdG interpreted the results and wrote the paper. Both authors read and approved the final manuscript.

## Supplementary Material

Additional File 1 **Total counts of repeats in the gene quintiles.** Total counts of repeats in the gene quintiles (the columns numbered 1 to 5 correspond to the first, second, third, fourth and fifth quintile of the gene), the fractions of the repeat counts in the different quintiles and the deviation from the expectancy values (i.e., 20%). Click here for file

Additional File 2 **Gene lists of the fungal species that have intragenic repeats 15 bp or longer.** Gene lists of the fungal species that have intragenic repeats 15 bp or longer (*Aspergillus flavus, Batrachochytrium dendrobatidis, Candida guilliermondii, Debaryomyces hansenii, Pyrenophora tritici-repentis and Schizosaccharomyces japonicus yfs275 *do not contain predicted genes with such long repeats). Since some genomes do not have unique gene identifiers, their names are complemented with the line number of the gene name in the original fasta file. Consecutive entries highlighted in green signify identical genes (a total of 4 are identified). Click here for file

Additional File 3 **Repertoire sizes of genes with different repeat lengths.** Gene repertoire sizes of the fungi, and the counts and fractions of genes that contain repeats of lengths five until greater than nine. Click here for file

## References

[B1] Smith HO, Tomb JF, Dougherty BA, Fleischmann RD, Venter JC (1995). Frequency and distribution of DNA uptake signal sequences in the Haemophilus influenzae Rd genome. Science.

[B2] Lawrence JG, Hendrickson H (2003). Lateral gene transfer: when will adolescence end?. Mol Microbiol.

[B3] Lawrence JG, Hendrickson H (2005). Genome evolution in bacteria: order beneath chaos. Curr Opin Microbiol.

[B4] Karlin S, Campbell AM, Mrazek J (1998). Comparative DNA analysis across diverse genomes. Annu Rev Genet.

[B5] Gentles AJ, Karlin S (2001). Genome-scale compositional comparisons in eukaryotes. Genome Res.

[B6] Bussemaker HJ, Li H, Siggia ED (2000). Regulatory element detection using a probabilistic segmentation model. Proc Int Conf Intell Syst Mol Biol.

[B7] Mrazek J, Guo X, Shah A (2007). Simple sequence repeats in prokaryotic genomes. Proc Natl Acad Sci U S A.

[B8] van Belkum A, Scherer S, van Alphen L, Verbrugh H (1998). Short-sequence DNA repeats in prokaryotic genomes. Microbiol Mol Biol Rev.

[B9] Karaoglu H, Lee CM, Meyer W (2005). Survey of simple sequence repeats in completed fungal genomes. Mol Biol Evol.

[B10] Coenye T, Vandamme P (2005). Characterization of mononucleotide repeats in sequenced prokaryotic genomes. DNA Res.

[B11] Hancock JM (1995). The contribution of slippage-like processes to genome evolution. J Mol Evol.

[B12] Ackermann M, Chao L (2006). DNA sequences shaped by selection for stability. PLoS Genet.

[B13] van Passel MW, Ochman H (2007). Selection on the genic location of disruptive elements. Trends Genet.

[B14] NCBI (2008). National Center for Biotechnology Information. http://www.ncbi.nlm.nih.gov/genomes/leuks.cgi.

[B15] James TY, Kauff F, Schoch CL, Matheny PB, Hofstetter V, Cox CJ, Celio G, Gueidan C, Fraker E, Miadlikowska J (2006). Reconstructing the early evolution of Fungi using a six-gene phylogeny. Nature.

[B16] Tatusov RL, Fedorova ND, Jackson JD, Jacobs AR, Kiryutin B, Koonin EV, Krylov DM, Mazumder R, Mekhedov SL, Nikolskaya AN (2003). The COG database: an updated version includes eukaryotes. BMC Bioinformatics.

[B17] Kuzniar A, van Ham RC, Pongor S, Leunissen JA (2008). The quest for orthologs: finding the corresponding gene across genomes. Trends Genet.

[B18] Toth G, Gaspari Z, Jurka J (2000). Microsatellites in different eukaryotic genomes: survey and analysis. Genome Res.

[B19] Malpertuy A, Dujon B, Richard GF (2003). Analysis of microsatellites in 13 hemiascomycetous yeast species: mechanisms involved in genome dynamics. J Mol Evol.

[B20] Kim TS, Booth JG, Gauch HG, Sun Q, Park J, Lee YH, Lee K (2008). Simple sequence repeats in Neurospora crassa: distribution, polymorphism and evolutionary inference. BMC Genomics.

[B21] Fitzpatrick DA, Logue ME, Stajich JE, Butler G (2006). A fungal phylogeny based on 42 complete genomes derived from supertree and combined gene analysis. BMC Evol Biol.

[B22] Akashi H, Gojobori T (2002). Metabolic efficiency and amino acid composition in the proteomes of Escherichia coli and Bacillus subtilis. Proc Natl Acad Sci USA.

[B23] Huntley MA, Clark AG (2007). Evolutionary analysis of amino acid repeats across the genomes of 12 Drosophila species. Mol Biol Evol.

[B24] Mirkin SM (2007). Expandable DNA repeats and human disease. Nature.

[B25] Kovtun IV, McMurray CT (2008). Features of trinucleotide repeat instability in vivo. Cell Res.

[B26] Kashi Y, King DG (2006). Simple sequence repeats as advantageous mutators in evolution. Trends Genet.

[B27] Bagshaw AT, Pitt JP, Gemmell NJ (2008). High frequency of microsatellites in S. cerevisiae meiotic recombination hotspots. BMC Genomics.

[B28] van Passel MW (2008). An intragenic distribution bias of DNA uptake sequences in Pasteurellaceae and Neisseriae. Biol Direct.

