# Supplementary material for: Mononucleotide repeats are asymmetrically distributed in fungal genes
Source: BMC Genomics. 2008 Dec 11;9:596. doi: 10.1186/1471-2164-9-596 (PMC2621210; doi:10.1186/1471-2164-9-596)
Supplement: Additional File 1 — Total counts of repeats in the gene quintiles. Total counts of repeats in the gene quintiles (the columns numbered 1 to 5 correspond to the first, second, third, fourth and fifth quintile of the gene), the fractions of the repeat counts in the different quintiles and the deviation from the expectancy values (i.e., 20%). [file 1471-2164-9-596-S1.docx]

**Additional file 1**.

File format: doc file

Title: Total counts of repeats in the gene quintiles

Description: Total counts of repeats in the gene quintiles (the columns numbered 1 to 5 correspond to the first, second, third, fourth and fifth quintile of the gene), the fractions of the repeat counts in the different quintiles and the deviation from the expectancy values (i.e., 20%).

|  |  |  |  | **Counts of repeats in gene quintiles (total)** | | | | |  |  | **Fraction of repeats in gene quintiles (%)** | | | | |  | **Deviation from expectancy (I.e., from 20%)** | | | | |
| --- | --- | --- | --- | --- | --- | --- | --- | --- | --- | --- | --- | --- | --- | --- | --- | --- | --- | --- | --- | --- | --- |
| **Species** | **# CDS** | **Significantly different (y/n)** | **Minimal repeat length (bp)** | **1** | **2** | **3** | **4** | **5** | **Total repeat count** |  | **1** | **2** | **3** | **4** | **5** |  | **1** | **2** | **3** | **4** | **5** |
| Ac | 129 | n | 8 | 38 | 29 | 20 | 20 | 25 | 132 |  | 28.8 | 22.0 | 15.2 | 15.2 | 18.9 |  | 8.8 | 2.0 | -4.8 | -4.8 | -1.1 |
| Afl | 138 | n | 8 | 33 | 36 | 27 | 23 | 21 | 140 |  | 23.6 | 25.7 | 19.3 | 16.4 | 15.0 |  | 3.6 | 5.7 | -0.7 | -3.6 | -5.0 |
| Afu | 219 | y | 8 | 67 | 37 | 32 | 40 | 63 | 239 |  | 28.0 | 15.5 | 13.4 | 16.7 | 26.4 |  | 8.0 | -4.5 | -6.6 | -3.3 | 6.4 |
| Anid | 186 | y | 8 | 63 | 34 | 38 | 28 | 34 | 197 |  | 32.0 | 17.3 | 19.3 | 14.2 | 17.3 |  | 12.0 | -2.7 | -0.7 | -5.8 | -2.7 |
| Anig | 106 | n | 8 | 33 | 15 | 22 | 28 | 24 | 122 |  | 27.0 | 12.3 | 18.0 | 23.0 | 19.7 |  | 7.0 | -7.7 | -2.0 | 3.0 | -0.3 |
| Ao | 176 | y | 8 | 56 | 41 | 18 | 30 | 34 | 179 |  | 31.3 | 22.9 | 10.1 | 16.8 | 19.0 |  | 11.3 | 2.9 | -9.9 | -3.2 | -1.0 |
| At | 617 | y | 7 | 164 | 120 | 118 | 103 | 155 | 660 |  | 24.8 | 18.2 | 17.9 | 15.6 | 23.5 |  | 4.8 | -1.8 | -2.1 | -4.4 | 3.5 |
| Bd | 212 | n | 9 | 36 | 54 | 52 | 42 | 34 | 218 |  | 16.5 | 24.8 | 23.9 | 19.3 | 15.6 |  | -3.5 | 4.8 | 3.9 | -0.7 | -4.4 |
| Bc | 305 | y | 8 | 64 | 60 | 58 | 45 | 93 | 320 |  | 20.0 | 18.8 | 18.1 | 14.1 | 29.1 |  | 0.0 | -1.3 | -1.9 | -5.9 | 9.1 |
| Ca_sc | 137 | y | 10 | 82 | 23 | 21 | 20 | 15 | 161 |  | 50.9 | 14.3 | 13.0 | 12.4 | 9.3 |  | 30.9 | -5.7 | -7.0 | -7.6 | -10.7 |
| Ca_wo | 94 | y | 10 | 50 | 17 | 13 | 12 | 12 | 104 |  | 48.1 | 16.3 | 12.5 | 11.5 | 11.5 |  | 28.1 | -3.7 | -7.5 | -8.5 | -8.5 |
| Cgu | 349 | y | 8 | 116 | 65 | 59 | 48 | 83 | 371 |  | 31.3 | 17.5 | 15.9 | 12.9 | 22.4 |  | 11.3 | -2.5 | -4.1 | -7.1 | 2.4 |
| Cl | 142 | y | 9 | 73 | 21 | 13 | 20 | 25 | 152 |  | 48.0 | 13.8 | 8.6 | 13.2 | 16.4 |  | 28.0 | -6.2 | -11.4 | -6.8 | -3.6 |
| Cp | 114 | y | 10 | 104 | 6 | 7 | 1 | 7 | 125 |  | 83.2 | 4.8 | 5.6 | 0.8 | 5.6 |  | 63.2 | -15.2 | -14.4 | -19.2 | -14.4 |
| Ct | 107 | y | 12 | 96 | 5 | 5 | 6 | 9 | 121 |  | 79.3 | 4.1 | 4.1 | 5.0 | 7.4 |  | 59.3 | -15.9 | -15.9 | -15.0 | -12.6 |
| Cgl | 198 | y | 9 | 42 | 28 | 23 | 47 | 63 | 203 |  | 20.7 | 13.8 | 11.3 | 23.2 | 31.0 |  | 0.7 | -6.2 | -8.7 | 3.2 | 11.0 |
| Ci_h | 261 | n | 8 | 55 | 55 | 65 | 52 | 48 | 275 |  | 20.0 | 20.0 | 23.6 | 18.9 | 17.5 |  | 0.0 | 0.0 | 3.6 | -1.1 | -2.6 |
| Ci_2394 | 302 | n | 8 | 64 | 68 | 73 | 64 | 47 | 316 |  | 20.3 | 21.5 | 23.1 | 20.3 | 14.9 |  | 0.3 | 1.5 | 3.1 | 0.3 | -5.1 |
| Ci_3703 | 278 | n | 8 | 64 | 56 | 73 | 47 | 52 | 292 |  | 21.9 | 19.2 | 25.0 | 16.1 | 17.8 |  | 1.9 | -0.8 | 5.0 | -3.9 | -2.2 |
| Ci_rs | 311 | n | 8 | 70 | 68 | 72 | 64 | 53 | 327 |  | 21.4 | 20.8 | 22.0 | 19.6 | 16.2 |  | 1.4 | 0.8 | 2.0 | -0.4 | -3.8 |
| Cp_3488 | 271 | n | 8 | 63 | 60 | 50 | 55 | 58 | 286 |  | 22.0 | 21.0 | 17.5 | 19.2 | 20.3 |  | 2.0 | 1.0 | -2.5 | -0.8 | 0.3 |
| Cp_silvereira | 269 | n | 8 | 64 | 52 | 55 | 53 | 55 | 279 |  | 22.9 | 18.6 | 19.7 | 19.0 | 19.7 |  | 2.9 | -1.4 | -0.3 | -1.0 | -0.3 |
| Cc | 105 | n | 8 | 21 | 30 | 16 | 20 | 21 | 108 |  | 19.4 | 27.8 | 14.8 | 18.5 | 19.4 |  | -0.6 | 7.8 | -5.2 | -1.5 | -0.6 |
| Cn | 254 | y | 8 | 99 | 45 | 39 | 44 | 57 | 284 |  | 34.9 | 15.8 | 13.7 | 15.5 | 20.1 |  | 14.9 | -4.2 | -6.3 | -4.5 | 0.1 |
| Dh | 113 | n | 9 | 25 | 24 | 20 | 18 | 31 | 118 |  | 21.2 | 20.3 | 16.9 | 15.3 | 26.3 |  | 1.2 | 0.3 | -3.1 | -4.7 | 6.3 |
| Fg | 113 | n | 8 | 30 | 25 | 19 | 18 | 23 | 115 |  | 26.1 | 21.7 | 16.5 | 15.7 | 20.0 |  | 6.1 | 1.7 | -3.5 | -4.3 | 0.0 |
| Fo | 114 | n | 8 | 33 | 19 | 23 | 17 | 24 | 116 |  | 28.4 | 16.4 | 19.8 | 14.7 | 20.7 |  | 8.4 | -3.6 | -0.2 | -5.3 | 0.7 |
| Fv | 105 | n | 8 | 27 | 17 | 22 | 23 | 19 | 108 |  | 25.0 | 15.7 | 20.4 | 21.3 | 17.6 |  | 5.0 | -4.3 | 0.4 | 1.3 | -2.4 |
| Hc | 114 | n | 9 | 22 | 22 | 22 | 20 | 29 | 115 |  | 19.1 | 19.1 | 19.1 | 17.4 | 25.2 |  | -0.9 | -0.9 | -0.9 | -2.6 | 5.2 |
| Le | 113 | y | 10 | 63 | 20 | 22 | 19 | 32 | 156 |  | 40.4 | 12.8 | 14.1 | 12.2 | 20.5 |  | 20.4 | -7.2 | -5.9 | -7.8 | 0.5 |
| Mg | 199 | y | 8 | 71 | 31 | 29 | 22 | 54 | 207 |  | 34.3 | 15.0 | 14.0 | 10.6 | 26.1 |  | 14.3 | -5.0 | -6.0 | -9.4 | 6.1 |
| Nf | 626 | y | 7 | 167 | 139 | 126 | 120 | 123 | 675 |  | 24.7 | 20.6 | 18.7 | 17.8 | 18.2 |  | 4.7 | 0.6 | -1.3 | -2.2 | -1.8 |
| Nc | 107 | n | 8 | 27 | 25 | 17 | 22 | 20 | 111 |  | 24.3 | 22.5 | 15.3 | 19.8 | 18.0 |  | 4.3 | 2.5 | -4.7 | -0.2 | -2.0 |
| Pb | 116 | n | 9 | 31 | 21 | 26 | 16 | 26 | 120 |  | 25.8 | 17.5 | 21.7 | 13.3 | 21.7 |  | 5.8 | -2.5 | 1.7 | -6.7 | 1.7 |
| Pg | 200 | n | 10 | 53 | 43 | 41 | 35 | 34 | 206 |  | 25.7 | 20.9 | 19.9 | 17.0 | 16.5 |  | 5.7 | 0.9 | -0.1 | -3.0 | -3.5 |
| Pt | 748 | y | 7 | 177 | 127 | 137 | 166 | 200 | 807 |  | 21.9 | 15.7 | 17.0 | 20.6 | 24.8 |  | 1.9 | -4.3 | -3.0 | 0.6 | 4.8 |
| Ro | 128 | y | 10 | 47 | 16 | 19 | 13 | 34 | 129 |  | 36.4 | 12.4 | 14.7 | 10.1 | 26.4 |  | 16.4 | -7.6 | -5.3 | -9.9 | 6.4 |
| Sce | 111 | n | 10 | 29 | 26 | 23 | 11 | 23 | 112 |  | 25.9 | 23.2 | 20.5 | 9.8 | 20.5 |  | 5.9 | 3.2 | 0.5 | -10.2 | 0.5 |
| Sj | 341 | y | 8 | 137 | 77 | 61 | 36 | 53 | 364 |  | 37.6 | 21.2 | 16.8 | 9.9 | 14.6 |  | 17.6 | 1.2 | -3.2 | -10.1 | -5.4 |
| So | 160 | y | 9 | 56 | 36 | 21 | 17 | 33 | 163 |  | 34.4 | 22.1 | 12.9 | 10.4 | 20.2 |  | 14.4 | 2.1 | -7.1 | -9.6 | 0.2 |
| Sp | 279 | y | 9 | 106 | 57 | 52 | 50 | 46 | 311 |  | 34.1 | 18.3 | 16.7 | 16.1 | 14.8 |  | 14.1 | -1.7 | -3.3 | -3.9 | -5.2 |
| Scl | 344 | y | 8 | 74 | 62 | 64 | 63 | 100 | 363 |  | 20.4 | 17.1 | 17.6 | 17.4 | 27.5 |  | 0.4 | -2.9 | -2.4 | -2.6 | 7.5 |
| Sn | 102 | n | 8 | 24 | 24 | 16 | 19 | 20 | 103 |  | 23.3 | 23.3 | 15.5 | 18.4 | 19.4 |  | 3.3 | 3.3 | -4.5 | -1.6 | -0.6 |
| Ur | 169 | y | 8 | 50 | 36 | 31 | 24 | 34 | 175 |  | 28.6 | 20.6 | 17.7 | 13.7 | 19.4 |  | 8.6 | 0.6 | -2.3 | -6.3 | -0.6 |
| Um | 449 | n | 7 | 113 | 104 | 88 | 76 | 97 | 478 |  | 23.6 | 21.8 | 18.4 | 15.9 | 20.3 |  | 3.6 | 1.8 | -1.6 | -4.1 | 0.3 |
| Va | 221 | y | 8 | 53 | 38 | 33 | 48 | 65 | 237 |  | 22.4 | 16.0 | 13.9 | 20.3 | 27.4 |  | 2.4 | -4.0 | -6.1 | 0.3 | 7.4 |
| Vd | 168 | y | 8 | 40 | 36 | 34 | 19 | 47 | 176 |  | 22.7 | 20.5 | 19.3 | 10.8 | 26.7 |  | 2.7 | 0.5 | -0.7 | -9.2 | 6.7 |
