# Supplementary material for: Mononucleotide repeats are asymmetrically distributed in fungal genes
Source: BMC Genomics. 2008 Dec 11;9:596. doi: 10.1186/1471-2164-9-596 (PMC2621210; doi:10.1186/1471-2164-9-596)
Supplement: Additional File 2 — Gene lists of the fungal species that have intragenic repeats 15 bp or longer. Gene lists of the fungal species that have intragenic repeats 15 bp or longer (Aspergillus flavus, Batrachochytrium dendrobatidis, Candida guilliermondii, Debaryomyces hansenii, Pyrenophora tritici-repentis and Schizosaccharomyces japonicus yfs275 do not contain predicted genes with such long repeats). Since some genomes do not have unique gene identifiers, their names are complemented with the line number of the gene name in the original fasta file. Consecutive entries highlighted in green signify identical genes (a total of 4 are identified). [file 1471-2164-9-596-S2.docx]

**Additional file 2**.

File format: doc file

Title: Gene lists of the fungal species that have intragenic repeats 15 bp or longer

Description: Gene lists of the fungal species that have intragenic repeats 15 bp or longer (*Aspergillus flavus, Batrachochytrium dendrobatidis, Candida guilliermondii, Debaryomyces hansenii, Pyrenophora tritici-repentis and Schizosaccharomyces japonicus yfs275* do not contain predicted genes with such long repeats). Since some genomes do not have unique gene identifiers, their names are complemented with the line number of the gene name in the original fasta file. Consecutive entries highlighted in green signify identical genes (a total of 4 are identified).

| **Species** | **Transcript name*** | **Repeat base and number** | **Relative intragenic position** | **Functional association [KOG classificaction]** |
| --- | --- | --- | --- | --- |
| *A. clavatus* | >null \| transcript encoding Aspergillus clavatus null (156 nt) 140345 | T23 | 0.25 |  |
|  | >null \| transcript encoding Aspergillus clavatus null (912 nt) 165848 | T24 | 0.93 | Arylalkylamine N-acetyltransferase[R] |
|  | >null \| transcript encoding Aspergillus clavatus null (240 nt) 114484 | C22 | 0.57 | Permease of the major facilitator superfamily [G] |
|  | >null \| transcript encoding Aspergillus clavatus null (2664 nt) 175161 | T22 | 0.81 | Serine/threonine protein kinase [T] |
| *A. fumigatus* | >null \| transcript encoding Aspergillus fumigatus null (864 nt) 78574 | G17 | 0.76 | Serine/threonine protein phosphatase 2A, regulatory subunit [T] |
|  | >null \| transcript encoding Aspergillus fumigatus null (1416 nt) 141019 | A37 | 0.74 | Zinc-binding oxidoreductase [CR] |
|  | >null \| transcript encoding Aspergillus fumigatus null (1089 nt) 43067 | G26 | 0.69 |  |
|  | >null \| transcript encoding Aspergillus fumigatus null (1353 nt) 125297 | C16 | 0.21 |  |
|  | >null \| transcript encoding Aspergillus fumigatus null (3822 nt) 231708 | T46 | 0.04 |  |
|  | >null \| transcript encoding Aspergillus fumigatus null (1503 nt) 195947 | C15 | 0.61 |  |
|  | >null \| transcript encoding Aspergillus fumigatus null (792 nt) 128956 | C17 | 0.30 |  |
|  | >null \| transcript encoding Aspergillus fumigatus null (957 nt) 236640 | T17 | 0.94 | Cytochrome P450 CYP4/CYP19/CYP26 subfamilies [QI] |
|  | >null \| transcript encoding Aspergillus fumigatus null (654 nt) 54902 | T16 | 0.66 |  |
|  | >null \| transcript encoding Aspergillus fumigatus null (2454 nt) 179217 | A18 | 0.81 |  |
|  | >null \| transcript encoding Aspergillus fumigatus null (765 nt) 86759 | G18 | 0.76 |  |
|  | >null \| transcript encoding Aspergillus fumigatus null (1599 nt) 229718 | C25 | 0.96 |  |
|  | >null \| transcript encoding Aspergillus fumigatus null (1599 nt) 229718 | T26 | 0.97 |  |
|  | >null \| transcript encoding Aspergillus fumigatus null (1428 nt) 69791 | G15 | 0.21 |  |
|  | >null \| transcript encoding Aspergillus fumigatus null (900 nt) 141651 | T39 | 0.51 |  |
|  | >null \| transcript encoding Aspergillus fumigatus null (2220 nt) 58129 | T39 | 0.98 | Cytochrome b5 [C] |
|  | >null \| transcript encoding Aspergillus fumigatus null (2028 nt) 160875 | C15 | 0.02 |  |
|  | >null \| transcript encoding Aspergillus fumigatus null (330 nt) 147261 | A16 | 0.76 |  |
|  | >null \| transcript encoding Aspergillus fumigatus null (1512 nt) 225941 | C18 | 0.71 |  |
| *A. nidulans* | >AN1T_02986 \| transcript encoding Aspergillus nidulans conserved hypothetical protein (1149 nt) | A24 | 0.70 |  |
| *A. niger* | >fge1_pg_C_4000838.1 \| transcript encoding Aspergillus niger fge1_pg_C_4000838 (891 nt) | A26 | 0.40 |  |
|  | >fge1_pg_C_9000307.1 \| transcript encoding Aspergillus niger fge1_pg_C_9000307 (1482 nt) | A20 | 0.97 |  |
|  | >fge1_pg_C_9000334.1 \| transcript encoding Aspergillus niger fge1_pg_C_9000334 (1056 nt) | T35 | 0.63 |  |
|  | >fge1_pg_C_15000127.1 \| transcript encoding Aspergillus niger fge1_pg_C_15000127 (417 nt) | A39 | 0.89 |  |
|  | >e_gw1_14.603.1 \| transcript encoding Aspergillus niger e_gw1_14.603 (1005 nt) | T50 | 0.86 |  |
|  | >fge1_pg_C_3000515.1 \| transcript encoding Aspergillus niger fge1_pg_C_3000515 (1740 nt) | C19 | 0.16 |  |
|  | >fge1_pg_C_2000445.1 \| transcript encoding Aspergillus niger fge1_pg_C_2000445 (498 nt) | A29 | 0.68 |  |
|  | >fge1_pg_C_7000238.1 \| transcript encoding Aspergillus niger fge1_pg_C_7000238 (783 nt) | A25 | 0.33 |  |
|  | >fge1_pg_C_14000271.1 \| transcript encoding Aspergillus niger fge1_pg_C_14000271 (417 nt) | A19 | 0.17 |  |
|  | >fge1_pg_C_7000031.1 \| transcript encoding Aspergillus niger fge1_pg_C_7000031 (324 nt) | A34 | 0.43 |  |
|  | >fge1_pg_C_13000033.1 \| transcript encoding Aspergillus niger fge1_pg_C_13000033 (1422 nt) | A40 | 0.34 |  |
|  | >fge1_pg_C_8000057.1 \| transcript encoding Aspergillus niger fge1_pg_C_8000057 (1764 nt) | A22 | 0.95 |  |
|  | >fge1_pg_C_9000097.1 \| transcript encoding Aspergillus niger fge1_pg_C_9000097 (909 nt) | A21 | 0.86 |  |
|  | >fge1_pg_C_9000052.1 \| transcript encoding Aspergillus niger fge1_pg_C_9000052 (1143 nt) | A18 | 0.58 |  |
|  | >e_gw1_9.635.1 \| transcript encoding Aspergillus niger e_gw1_9.635 (1407 nt) | T24 | 0.95 | a) Cytochrome P450 CYP2 subfamily [Q]  b) Cytochrome P450 CYP4/CYP19/CYP26 subfamilies [QI]  c) Cytochrome P450 CYP3/CYP5/CYP6/CYP9 subfamilies [Q] |
|  | >fge1_pg_C_9000003.1 \| transcript encoding Aspergillus niger fge1_pg_C_9000003 (1266 nt) | A35 | 0.97 |  |
|  | >fge1_pg_C_19000186.1 \| transcript encoding Aspergillus niger fge1_pg_C_19000186 (909 nt) | C16 | 0.07 |  |
|  | >fge1_pg_C_11000006.1 \| transcript encoding Aspergillus niger fge1_pg_C_11000006 (567 nt) | A18 | 0.29 |  |
| *A. oryzae* | >null \| transcript encoding Aspergillus oryzae null (1731 nt) 209856 | A15 | 0.95 |  |
|  | >null \| transcript encoding Aspergillus oryzae null (1584 nt) 112716 | T18 | 0.07 |  |
|  | >null \| transcript encoding Aspergillus oryzae null (1497 nt) 21289 | A17 | 0.18 |  |
|  | >null \| transcript encoding Aspergillus oryzae null (1845 nt) 174795 | G21 | 0.03 | Transcriptional activator FOSB/c-Fos and related bZIP transcription factors [K] |
|  | >null \| transcript encoding Aspergillus oryzae null (1584 nt) 30596 | A29 | 0.23 |  |
|  | >null \| transcript encoding Aspergillus oryzae null (990 nt) 5132 | T17 | 0.62 |  |
|  | >null \| transcript encoding Aspergillus oryzae null (753 nt) 117495 | A18 | 0.12 |  |
|  | >null \| transcript encoding Aspergillus oryzae null (573 nt) 165944 | A16 | 0.30 | Iron/ascorbate family oxidoreductases [QR] |
| *A. terreus* | >ATET_03009 \| transcript encoding Aspergillus terreus predicted protein (1053 nt) | A38 | 0.54 |  |
|  | >ATET_09934 \| transcript encoding Aspergillus terreus predicted protein (555 nt) | C18 | 0.31 | Tryptophan synthase beta chain [E] |
|  | >ATET_00185 \| transcript encoding Aspergillus terreus predicted protein (1437 nt) | A68 | 0.77 |  |
| *B. cinerea* | >BC1T_15693 \| transcript encoding Botrytis cinerea predicted protein (249 nt) | A16 | 0.23 |  |
|  | >BC1T_08371 \| transcript encoding Botrytis cinerea hypothetical protein (1095 nt) | G17 | 0.95 | Predicted membrane protein [S] |
|  | >BC1T_13522 \| transcript encoding Botrytis cinerea hypothetical protein (3285 nt) | G16 | 0.68 | Guanine nucleotide exchange factor [U] |
|  | >BC1T_03204 \| transcript encoding Botrytis cinerea predicted protein (354 nt) | G17 | 0.46 |  |
|  | >BC1T_13838 \| transcript encoding Botrytis cinerea hypothetical protein (456 nt) | G15 | 0.91 | Predicted mitochondrial ribosomal protein L23 [J] |
|  | >BC1T_10238 \| transcript encoding Botrytis cinerea predicted protein (354 nt) | G19 | 0.83 |  |
| *C. albicans sc5314* | >orf19_4636.1 \| transcript encoding Candida albicans SC5314 orf19.4636 (423 nt) | G15 | 0.22 |  |
|  | >orf19_7348.1 \| transcript encoding Candida albicans SC5314 orf19.7348 (321 nt) | T17 | 0.56 |  |
|  | >orf19_3229.1 \| transcript encoding Candida albicans SC5314 orf19.3229 (306 nt) | T16 | 0.65 |  |
|  | >orf19_6341.1 \| transcript encoding Candida albicans SC5314 orf19.6341 (915 nt) | A18 | 0.48 |  |
|  | >orf19_6575.1 \| transcript encoding Candida albicans SC5314 orf19.6575 (327 nt) | A15 | 0.35 |  |
|  | >orf19_2539.1 \| transcript encoding Candida albicans SC5314 orf19.2539 (336 nt) | T15 | 0.68 |  |
|  | >orf19_7194.1 \| transcript encoding Candida albicans SC5314 orf19.7194 (927 nt) | A15 | 0.06 |  |
|  | >orf19_6534.1 \| transcript encoding Candida albicans SC5314 orf19.6534 (471 nt) | T15 | 0.66 |  |
|  | >orf19_322.1 \| transcript encoding Candida albicans SC5314 orf19.322 (435 nt) | A16 | 0.74 |  |
|  | >orf19_3196.1 \| transcript encoding Candida albicans SC5314 orf19.3196 (303 nt) | T15 | 0.14 |  |
|  | >orf19_3479.1 \| transcript encoding Candida albicans SC5314 orf19.3479 (381 nt) | A22 | 0.20 |  |
| *C. albicans wo1* | >CAWT_05718 \| transcript encoding Candida albicans WO1 conserved hypothetical protein (927 nt) | A15 | 0.06 |  |
|  | >CAWT_04548 \| transcript encoding Candida albicans WO1 conserved hypothetical protein (1476 nt) | A17 | 0.02 | Pseudouridylate synthase [J] |
|  | >CAWT_02878 \| transcript encoding Candida albicans WO1 conserved hypothetical protein (309 nt) | T17 | 0.58 |  |
|  | >CAWT_03900 \| transcript encoding Candida albicans WO1 conserved hypothetical protein (711 nt) | T15 | 0.23 |  |
|  | >CAWT_00687 \| transcript encoding Candida albicans WO1 conserved hypothetical protein (396 nt) | T16 | 0.47 |  |
|  | >CAWT_06122 \| transcript encoding Candida albicans WO1 conserved hypothetical protein (405 nt) | A16 | 0.33 |  |
|  | >CAWT_00161 \| transcript encoding Candida albicans WO1 5-amino-6-(5-phosphoribosylamino)uracil reductase (909 nt) | A15 | 0.48 |  |
|  | >CAWT_05656 \| transcript encoding Candida albicans WO1 hypothetical protein similar to mitochondrial Complex I (384 nt) | T17 | 0.17 |  |
|  | >CAWT_05537 \| transcript encoding Candida albicans WO1 conserved hypothetical protein (471 nt) | T15 | 0.66 |  |
|  | >CAWT_05504 \| encoding Candida albicans WO1 conserved hypothetical protein (327 nt) | A16 | 0.35 |  |
|  | >CAWT_03964 \| transcript encoding Candida albicans WO1 predicted protein (336 nt) | T15 | 0.54 |  |
| *C. lusitaniae* | >CLUT_01742 \| transcript encoding Candida lusitaniae hypothetical protein (936 nt) | T28 | 0.06 |  |
|  | >CLUT_00566 \| transcript encoding Candida lusitaniae hypothetical protein (1083 nt) | A25 | 0.04 |  |
|  | >CLUT_00859 \| transcript encoding Candida lusitaniae predicted protein (498 nt) | T26 | 0.56 |  |
|  | >CLUT_05752 \| transcript encoding Candida lusitaniae eukaryotic translation initiation factor 5A (603 nt) | T15 | 0.10 | Translation initiation factor 5A (eIF-5A) [E] |
|  | >CLUT_03493 \| transcript encoding Candida lusitaniae hypothetical protein (723 nt) | A18 | 0.08 |  |
|  | >CLUT_04090 \| transcript encoding Candida lusitaniae hypothetical protein (1536 nt) | T15 | 0.07 |  |
|  | >CLUT_04370 \| transcript encoding Candida lusitaniae hypothetical protein (705 nt) | A19 | 0.16 |  |
|  | >CLUT_05336 \| transcript encoding Candida lusitaniae predicted protein (657 nt) | A30 | 0.18 |  |
|  | >CLUT_05008 \| transcript encoding Candida lusitaniae predicted protein (1488 nt) | T16 | 0.02 | Aspartyl protease [O] |
|  | >CLUT_01620 \| transcript encoding Candida lusitaniae hypothetical protein similar to mycophenolic acid-resistant inosine-5'-monophosphate dehydrogenase (552 nt) | T15 | 0.87 | IMP dehydrogenase/GMP reductase [F] |
|  | >CLUT_00425 \| transcript encoding Candida lusitaniae predicted protein (468 nt) | A18 | 0.86 |  |
|  | >CLUT_05553 \| transcript encoding Candida lusitaniae predicted protein (546 nt) 136996 | T33 | 0.85 |  |
|  | >CLUT_03093 \| transcript encoding Candida lusitaniae hypothetical protein (1725 nt) | T17 | 0.09 | Predicted N6-adenine RNA methylase [A] |
| *C. tropicalis* | >CTRT_00029 \| transcript encoding Candida tropicalis predicted protein (681 nt) | A15 | 0.03 |  |
|  | >CTRT_00029 \| transcript encoding Candida tropicalis predicted protein (681 nt) | A16 | 0.1 |  |
|  | >CTRT_00065 \| transcript encoding Candida tropicalis predicted protein (390 nt) | A15 | 0.72 |  |
|  | >CTRT_00074 \| transcript encoding Candida tropicalis conserved hypothetical protein (612 nt) | A18 | 0.1 |  |
|  | >CTRT_00287 \| transcript encoding Candida tropicalis conserved hypothetical protein (1923 nt) | T17 | 0.05 | Aminoacylase ACY1 and related metalloexopeptidases [E] |
|  | >CTRT_00317 \| transcript encoding Candida tropicalis hypothetical protein similar to potential tRNA ribose methyltransferase (4305 nt) | T17 | 0.03 | RNA Methylase, SpoU family [A] |
|  | >CTRT_00412 \| transcript encoding Candida tropicalis conserved hypothetical protein (954 nt) | T27 | 0.09 | N-acetylglucosaminyltransferase complex, subunit PIG-C/GPI2, required for phosphatidylinositol biosynthesis [I] |
|  | >CTRT_00449 \| transcript encoding Candida tropicalis histone H2A variant (582 nt) | T15 | 0.19 | Histone 2A [B] |
|  | >CTRT_00539 \| transcript encoding Candida tropicalis predicted protein (2880 nt) | A18 | 0.03 |  |
|  | >CTRT_01182 \| transcript encoding Candida tropicalis conserved hypothetical protein (1968 nt) | T16 | 0.04 | Uncharacterized conserved protein [S] |
|  | >CTRT_01596 \| transcript encoding Candida tropicalis conserved hypothetical protein (801 nt) | A15 | 0.05 | Uncharacterized conserved protein [S] |
|  | >CTRT_01659 \| transcript encoding Candida tropicalis conserved hypothetical protein (879 nt) | A15 | 0.04 | Uncharacterized conserved protein [S] |
|  | >CTRT_01949 \| transcript encoding Candida tropicalis hypothetical protein similar to DNA mismatch repair protein MutS (3048 nt) | T17 | 0.05 | Mismatch repair MSH3 [L] |
|  | >CTRT_02379 \| transcript encoding Candida tropicalis predicted protein (390 nt) | A22 | 0.27 |  |
|  | >CTRT_02380 \| transcript encoding Candida tropicalis predicted protein (360 nt) | T22 | 0.68 |  |
|  | >CTRT_02686 \| transcript encoding Candida tropicalis conserved hypothetical protein (570 nt) | A16 | 0.12 | Uncharacterized conserved protein [S] |
|  | >CTRT_02776 \| transcript encoding Candida tropicalis conserved hypothetical protein (846 nt) | A20 | 0.09 |  |
|  | >CTRT_02825 \| transcript encoding Candida tropicalis hypothetical protein similar to potential low affinity Ca2+ influx system membrane protein (963 nt) | T15 | 0.18 |  |
|  | >CTRT_02886 \| transcript encoding Candida tropicalis hypothetical protein similar to dead box helicase (1818 nt) | A16 | 0.03 | RNA helicase [A] |
|  | >CTRT_03050 \| transcript encoding Candida tropicalis conserved hypothetical protein (537 nt) | A20 | 0.07 | GPI transamidase complex, GPI17/PIG-S component, involved in glycosylphosphatidylinositol anchor biosynthesis [MO] |
|  | >CTRT_03068 \| transcript encoding Candida tropicalis conserved hypothetical protein (540 nt) | T16 | 0.05 |  |
|  | >CTRT_03444 \| transcript encoding Candida tropicalis predicted protein (1998 nt) | A20 | 0.03 | a) FOG: Leucine rich repeat[R]  b) Membrane glycoprotein LIG-1[T]  c) FOG: Toll/interleukin receptor and related proteins containing LRR and TIR repeats[T] |
|  | >CTRT_03551 \| transcript encoding Candida tropicalis predicted protein (783 nt) | T16 | 0.11 | Uncharacterized conserved protein[S] |
|  | >CTRT_03696 \| transcript encoding Candida tropicalis conserved hypothetical protein (1095 nt) | A15 | 0.03 | Uncharacterized conserved protein[S] |
|  | >CTRT_03729 \| transcript encoding Candida tropicalis hypothetical protein similar to CaNAG3 (1818 nt) | A17 | 0.02 | Synaptic vesicle transporter SVOP and related transporters (major facilitator superfamily)[R] |
|  | >CTRT_03748 \| transcript encoding Candida tropicalis conserved hypothetical protein (1263 nt) | A26 | 0.04 |  |
|  | >CTRT_03781 \| transcript encoding Candida tropicalis predicted protein (375 nt) | A17 | 0.4 |  |
|  | >CTRT_03832 \| transcript encoding Candida tropicalis predicted protein (387 nt) | T15 | 0.91 |  |
|  | >CTRT_03860 \| transcript encoding Candida tropicalis tubulin gamma chain (1611 nt) | T15 | 0.01 | Gamma tubulin [Z] |
|  | >CTRT_03967 \| transcript encoding Candida tropicalis predicted protein (990 nt) | T15 | 0.12 |  |
|  | >CTRT_04078 \| transcript encoding Candida tropicalis predicted protein (1713 nt) | T17 | 0.03 |  |
|  | >CTRT_04308 \| transcript encoding Candida tropicalis conserved hypothetical protein (765 nt) | A17 | 0.04 | Phosphoglycerate mutase [G] |
|  | >CTRT_04510 \| transcript encoding Candida tropicalis hypothetical protein similar to potential GINS DNA replication initiation complex subunit (798 nt) | A24 | 0.16 | Uncharacterized conserved protein [S] |
|  | >CTRT_04570 \| transcript encoding Candida tropicalis conserved hypothetical protein (1815 nt) | A15 | 0.03 |  |
|  | >CTRT_04765 \| transcript encoding Candida tropicalis conserved hypothetical protein (1065 nt) | T15 | 0.05 | Exosomal 3'-5' exoribonuclease complex, subunit ski4 (Csl4) [J] |
|  | >CTRT_04860 \| transcript encoding Candida tropicalis conserved hypothetical protein (2157 nt) | A15 | 0.1 | DNA repair exonuclease MRE11 [L] |
|  | >CTRT_05148 \| transcript encoding Candida tropicalis hypothetical protein similar to potential MATE family drug/sodium antiporter (1929 nt) | A16 | 0.03 | Uncharacterized membrane protein, predicted efflux pump [R] |
|  | >CTRT_05569 \| transcript encoding Candida tropicalis conserved hypothetical protein (1464 nt) | A17 | 0.03 |  |
|  | >CTRT_05873 \| transcript encoding Candida tropicalis predicted protein (444 nt) | T15 | 0.8 |  |
|  | >CTRT_06080 \| transcript encoding Candida tropicalis predicted protein (396 nt) | T16 | 0.93 |  |
|  | >CTRT_06111 \| transcript encoding Candida tropicalis conserved hypothetical protein (2250 nt) | A27 | 0.02 | Cytosolic Ca2+-dependent cysteine protease (calpain), large subunit (EF-Hand protein superfamily) [OT] |
| *C. parapsilosis* | >CPAG_00154.1 \| transcript encoding Candida parapsilosis CPAG_00154 (498 nt) | A19 | 0.11 | Molecular chaperone Prefoldin, subunit 2 [O] |
|  | >CPAG_00350.1 \| transcript encoding Candida parapsilosis CPAG_00350 (558 nt) | A16 | 0.15 | 60S ribosomal protein L14/L17/L23 [J] |
|  | >CPAG_00557.1 \| transcript encoding Candida parapsilosis CPAG_00557 (957 nt) | A15 | 0.02 | Chromatin remodeling protein, contains PHD Zn-finger [B] |
|  | >CPAG_00638.1 \| transcript encoding Candida parapsilosis CPAG_00638 (1239 nt) | T19 | 0.06 |  |
|  | >CPAG_00666.1 \| transcript encoding Candida parapsilosis CPAG_00666 (906 nt) | T17 | 0.03 | Pyridoxal/pyridoxine/pyridoxamine kinase [H] |
|  | >CPAG_00709.1 \| transcript encoding Candida parapsilosis CPAG_00709 (1404 nt) | T15 | 0.06 | S-adenosylmethionine synthetase [H] |
|  | >CPAG_01053.1 \| transcript encoding Candida parapsilosis CPAG_01053 (969 nt) | A18 | 0.24 | DNA-binding protein C1D involved in regulation of double-strand break repair [L] |
|  | >CPAG_01308.1 \| transcript encoding Candida parapsilosis CPAG_01308 (1572 nt) | A16 | 0.04 | ATP-dependent RNA helicase [A] |
|  | >CPAG_01967.1 \| transcript encoding Candida parapsilosis CPAG_01967 (567 nt) | T16 | 0.04 | Nucleoside diphosphate kinase [F] |
|  | >CPAG_01987.1 \| transcript encoding Candida parapsilosis CPAG_01987 (2475 nt) | A16 | 0.01 | WD40-repeat-containing subunit of the 18S rRNA processing complex [A] |
|  | >CPAG_01999.1 \| transcript encoding Candida parapsilosis CPAG_01999 (495 nt) | T21 | 0.79 |  |
|  | >CPAG_02200.1 \| transcript encoding Candida parapsilosis CPAG_02200 (5472 nt) | A15 | 0 | Uncharacterized conserved protein [S] |
|  | >CPAG_02255.1 \| transcript encoding Candida parapsilosis CPAG_02255 (1650 nt) | C18 | 0.04 | Members of tubulin/FtsZ family [Z] |
|  | >CPAG_02371.1 \| transcript encoding Candida parapsilosis CPAG_02371 (501 nt) | T15 | 0.14 | Uncharacterized conserved protein NOF (Neighbor of FAU) [S] |
|  | >CPAG_02419.1 \| transcript encoding Candida parapsilosis CPAG_02419 (729 nt) | G16 | 0.18 |  |
|  | >CPAG_02599.1 \| transcript encoding Candida parapsilosis CPAG_02599 (3762 nt) | A18 | 0.02 | GTP-binding protein AARP2 involved in 40S ribosome biogenesis [J] |
|  | >CPAG_02719.1 \| transcript encoding Candida parapsilosis CPAG_02719 (2601 nt) | A15 | 0.01 | DNA-binding protein YL1 and related proteins [R] |
|  | >CPAG_03017.1 \| transcript encoding Candida parapsilosis CPAG_03017 (456 nt) | T18 | 0.43 |  |
|  | >CPAG_03037.1 \| transcript encoding Candida parapsilosis CPAG_03037 (984 nt) | A15 | 0.04 | Isoamyl acetate-hydrolyzing esterase and related enzymes [R] |
|  | >CPAG_03457.1 \| transcript encoding Candida parapsilosis CPAG_03457 (2268 nt) | A18 | 0.06 | Prolyl-tRNA synthetase [J] |
|  | >CPAG_03559.1 \| transcript encoding Candida parapsilosis CPAG_03559 (1170 nt) | A18 | 0.05 | Uncharacterized conserved protein, contains CCCH-type Zn-finger[R] |
|  | >CPAG_03564.1 \| transcript encoding Candida parapsilosis CPAG_03564 (1014 nt) | T17 | 0.05 |  |
|  | >CPAG_03715.1 \| transcript encoding Candida parapsilosis CPAG_03715 (759 nt) | A16 | 0.05 | Farnesyl cysteine-carboxyl methyltransferase[O] |
|  | >CPAG_03716.1 \| transcript encoding Candida parapsilosis CPAG_03716 (771 nt) | T16 | 0.06 | Farnesyl cysteine-carboxyl methyltransferase[O] |
|  | >CPAG_03723.1 \| transcript encoding Candida parapsilosis CPAG_03723 (987 nt) | A16 | 0.03 |  |
|  | >CPAG_03804.1 \| transcript encoding Candida parapsilosis CPAG_03804 (2868 nt) | A17 | 0.04 | Threonyl-tRNA synthetase[J] |
|  | >CPAG_03968.1 \| transcript encoding Candida parapsilosis CPAG_03968 (3789 nt) | A15 | 0.03 | Nucleolar RNA-associated protein (NRAP)[S] |
|  | >CPAG_04523.1 \| transcript encoding Candida parapsilosis CPAG_04523 (1584 nt) | T15 | 0.01 | Folylpolyglutamate synthase[H] |
|  | >CPAG_05128.1 \| transcript encoding Candida parapsilosis CPAG_05128 (1254 nt) | T17 | 0.03 | Predicted undecaprenyl diphosphate synthase[I] |
|  | >CPAG_05196.1 \| transcript encoding Candida parapsilosis CPAG_05196 (2199 nt) | T17 | 0.01 | Thiamine pyrophosphate-requiring enzyme[EH] |
|  | >CPAG_05547.1 \| transcript encoding Candida parapsilosis CPAG_05547 (1713 nt) | A20 | 0.04 | ATP sulfurylase (sulfate adenylyltransferase)[P] |
|  | >CPAG_05570.1 \| transcript encoding Candida parapsilosis CPAG_05570 (612 nt) | T16 | 0.88 | Riboflavin kinase[H] |
|  | >CPAG_05639.1 \| transcript encoding Candida parapsilosis CPAG_05639 (678 nt) | A15 | 0.08 | Threonyl-tRNA synthetase[J] |
| *C. globosum* | >CHGT_02130 \| transcript encoding Chaetomium globosum hypothetical protein (3717 nt) | C15 | 0.70 | DNA-binding centromere protein B (CENP-B) [BD] |
|  | >CHGT_09877 \| transcript encoding Chaetomium globosum conserved hypothetical protein (813 nt) | C17 | 0.45 | Sortilin and related receptors [R] |
|  | >CHGT_07030 \| transcript encoding Chaetomium globosum hypothetical protein (3009 nt) | G17 | 0.89 | FOG: Zn-finger [R] |
|  | >CHGT_02843 \| transcript encoding Chaetomium globosum hypothetical protein (4581 nt) | G18 | 0.97 |  |
|  | >CHGT_08011 \| transcript encoding Chaetomium globosum predicted protein (462 nt) | C15 | 0.12 |  |
|  | >CHGT_02699 \| transcript encoding Chaetomium globosum predicted protein (726 nt) | G16 | 0.67 |  |
| *C. immitis h538.4* | >CIHT_03302 \| transcript encoding Coccidioides immitis H538.4 predicted protein (351 nt) | T19 | 0.15 |  |
|  | >CIHT_04135 \| transcript encoding Coccidioides immitis H538.4 predicted protein (597 nt) | G16 | 0.54 |  |
|  | >CIHT_00878 \| transcript encoding Coccidioides immitis H538.4 predicted protein (417 nt) | A18 | 0.33 |  |
|  | >CIHT_07554 \| transcript encoding Coccidioides immitis H538.4 hypothetical protein (402 nt) | T21 | 0.45 |  |
|  | >CIHT_06842 \| transcript encoding Coccidioides immitis H538.4 predicted protein (354 nt) | T16 | 0.83 |  |
| *C. immitis rmscc 2394* | >CIRT_02347 \| transcript encoding Coccidioides immitis RMSCC 2394 hypothetical protein (408 nt) | T27 | 0.45 |  |
|  | >CIRT_07378 \| transcript encoding Coccidioides immitis RMSCC 2394 predicted protein (351 nt) | T18 | 0.10 |  |
|  | >CIRT_07215 \| transcript encoding Coccidioides immitis RMSCC 2394 predicted protein (594 nt) | C19 | 0.40 |  |
| *C. immitis rmscc 3703* | >CIST_04957 \| transcript encoding Coccidioides immitis RMSCC 3703 predicted protein (414 nt) | C16 | 0.89 | 60s ribosomal protein L19 [J] |
|  | >CIST_08731 \| transcript encoding Coccidioides immitis RMSCC 3703 predicted protein (357 nt) | C22 | 0.79 |  |
|  | >CIST_06965 \| transcript encoding Coccidioides immitis RMSCC 3703 predicted protein (441 nt) | T16 | 0.53 |  |
|  | >CIST_03143 \| transcript encoding Coccidioides immitis RMSCC 3703 predicted protein (429 nt) | T16 | 0.05 |  |
|  | >CIST_00164 \| transcript encoding Coccidioides immitis RMSCC 3703 predicted protein (351 nt) | T18 | 0.10 |  |
|  | >CIST_03385 \| transcript encoding Coccidioides immitis RMSCC 3703 predicted protein (381 nt) | T20 | 0.11 |  |
|  | >CIST_04306 \| transcript encoding Coccidioides immitis RMSCC 3703 hypothetical protein (408 nt) | T27 | 0.45 |  |
| *C. immitis rs* | >CIMT_10100 \| transcript encoding Coccidioides immitis RS hypothetical protein (408 nt) | T27 | 0.45 |  |
|  | >CIMT_04968 \| transcript encoding Coccidioides immitis RS predicted protein (441 nt) | T16 | 0.53 |  |
|  | >CIMT_06755 \| transcript encoding Coccidioides immitis RS predicted protein (417 nt) | A18 | 0.33 |  |
|  | >CIMT_01219 \| transcript encoding Coccidioides immitis RS predicted protein (429 nt) | T16 | 0.05 |  |
|  | >CIMT_03614 \| transcript encoding Coccidioides immitis RS predicted protein (351 nt) | T18 | 0.10 |  |
|  | >CIMT_04290 \| transcript encoding Coccidioides immitis RS homoserine kinase (555 nt) | A16 | 0.88 | Homoserine kinase [E] |
|  | >CIMT_07215 \| transcript encoding Coccidioides immitis RS lipase 2 (1092 nt) | T18 | 0.42 | Arylacetamide deacetylase [V] |
|  | >CIMT_05383 \| transcript encoding Coccidioides immitis RS cytochrome c (513 nt) | T17 | 0.24 |  |
| *C. posadasii rmscc 3488* | >CPAT_05491 \| transcript encoding Coccidioides posadasii RMSCC 3488 predicted protein (339 nt) | G21 | 0.64 |  |
|  | >CPAT_07478 \| transcript encoding Coccidioides posadasii RMSCC 3488 conserved hypothetical protein (393 nt) | G15 | 0.52 |  |
|  | >CPAT_09829 \| transcript encoding Coccidioides posadasii RMSCC 3488 predicted protein (441 nt) | A15 | 0.32 |  |
|  | >CPAT_01954 \| transcript encoding Coccidioides posadasii RMSCC 3488 predicted protein (363 nt) | T15 | 0.16 |  |
|  | >CPAT_03034 \| transcript encoding Coccidioides posadasii RMSCC 3488 ATP citrate lyase subunit (1434 nt) | A17 | 0.35 | ATP-citrate lyase [C] |
|  | >CPAT_03034 \| transcript encoding Coccidioides posadasii RMSCC 3488 ATP citrate lyase subunit (1434 nt) | C26 | 0.33 | ATP-citrate lyase [C] |
|  | >CPAT_04230 \| transcript encoding Coccidioides posadasii RMSCC 3488 predicted protein (498 nt) | C17 | 0.73 |  |
|  | >CPAT_01873 \| transcript encoding Coccidioides posadasii RMSCC 3488 predicted protein (318 nt) | G15 | 0.59 |  |
|  | >CPAT_06749 \| transcript encoding Coccidioides posadasii RMSCC 3488 hypothetical protein (195 nt) | A16 | 0.46 |  |
| *C. posadasii str. silveira* | >CPST_02954 \| transcript encoding Coccidioides posadasii str. Silveira predicted protein (333 nt) | T17 | 0.15 |  |
|  | >CPST_07917 \| transcript encoding Coccidioides posadasii str. Silveira predicted protein (441 nt) | A15 | 0.32 |  |
|  | >CPST_02147 \| transcript encoding Coccidioides posadasii str. Silveira predicted protein (339 nt) | C19 | 0.59 |  |
|  | >CPST_06459 \| transcript encoding Coccidioides posadasii str. Silveira predicted protein (534 nt) | A24 | 0.43 |  |
| *C. cinereus* | >CC1T_11188 \| transcript encoding Coprinus cinereus hypothetical protein (387 nt) | G16 | 0.68 |  |
|  | >CC1T_03031 \| transcript encoding Coprinus cinereus predicted protein (159 nt) | C15 | 0.74 |  |
|  | >CC1T_09988 \| transcript encoding Coprinus cinereus predicted protein (474 nt) | C18 | 0.28 |  |
|  | >CC1T_10831 \| transcript encoding Coprinus cinereus predicted protein (471 nt) | C16 | 0.04 |  |
| *C. neoformans h99* | >CNAT_05579 \| transcript encoding Cryptococcus neoformans (H99) hypothetical protein (2268 nt) | A29 | 0.97 | RNA-binding protein RBM5 and related proteins, contain G-patch and RRM domains [R] |
| *F. graminearum* | >FGST_07263 \| transcript encoding Fusarium graminearum predicted protein (1167 nt) | C17 | 0.73 |  |
| *F. oxysporum f. sp. lycopersici* | >FOXT_08685 \| transcript encoding Fusarium oxysporum f. sp. lycopersici predicted protein (372 nt) | C16 | 0.12 |  |
|  | >FOXT_06920 \| transcript encoding Fusarium oxysporum f. sp. lycopersici conserved hypothetical protein (180 nt) | G15 | 0.65 |  |
| *F. verticillioides* | >FVET_05955 \| transcript encoding Fusarium verticillioides predicted protein (1914 nt) | G20 | 0.65 |  |
| *H. capsulatum nam1* | >HCAT_01889 \| transcript encoding Histoplasma capsulatum NAm1 predicted protein (555 nt) | A15 | 0.19 | Density-regulated protein related to translation initiation factor 1 (eIF-1/SUI1) [R] |
|  | >HCAT_04429 \| transcript encoding Histoplasma capsulatum NAm1 predicted protein (1356 nt) | T16 | 0.50 | SPRY domain-containing proteins [R] |
|  | >HCAT_07184 \| transcript encoding Histoplasma capsulatum NAm1 predicted protein (1593 nt) | G16 | 0.78 |  |
|  | >HCAT_06672 \| transcript encoding Histoplasma capsulatum NAm1 predicted protein (474 nt) | T37 | 0.09 |  |
|  | >HCAT_04525 \| transcript encoding Histoplasma capsulatum NAm1 predicted protein (1887 nt) | G15 | 0.98 | Nuclear porin [Y] |
|  | >HCAT_02129 \| transcript encoding Histoplasma capsulatum NAm1 predicted protein (1311 nt) | G15 | 0.91 |  |
|  | >HCAT_07130 \| transcript encoding Histoplasma capsulatum NAm1 predicted protein (903 nt) | G15 | 0.88 | Predicted dehydrogenase [R] |
|  | >HCAT_06596 \| transcript encoding Histoplasma capsulatum NAm1 predicted protein (2373 nt) | C15 | 0.19 | Casein kinase (serine/threonine/tyrosine protein kinase) [T] |
| *L. elongisporus* | >LELT_00080 \| transcript encoding Lodderomyces elongisporus predicted protein (540 nt) | A16 | 0.79 |  |
|  | >LELT_00956 \| transcript encoding Lodderomyces elongisporus conserved hypothetical protein (525 nt) | A21 | 0.13 |  |
|  | >LELT_01295 \| transcript encoding Lodderomyces elongisporus conserved hypothetical protein (1719 nt) | A17 | 0.06 | Putative alpha 1,2 mannosyltransferase [G] |
|  | >LELT_02450 \| transcript encoding Lodderomyces elongisporus predicted protein (483 nt) | T17 | 0.51 |  |
|  | >LELT_02933 \| transcript encoding Lodderomyces elongisporus predicted protein (1629 nt) | A20 | 0.02 |  |
|  | >LELT_03066 \| transcript encoding Lodderomyces elongisporus hypothetical protein (477 nt) | T17 | 0.13 |  |
|  | >LELT_03919 \| transcript encoding Lodderomyces elongisporus predicted protein (645 nt) | A15 | 0.88 |  |
|  | >LELT_04487 \| transcript encoding Lodderomyces elongisporus conserved hypothetical protein (924 nt) | A17 | 0.06 |  |
|  | >LELT_04889 \| transcript encoding Lodderomyces elongisporus conserved hypothetical protein (2163 nt) | T16 | 0.11 | Vacuolar sorting protein VPS45/Stt10 (Sec1 family) [U] |
|  | >LELT_04929 \| transcript encoding Lodderomyces elongisporus conserved hypothetical protein (630 nt) | A16 | 0.91 | Pleiotropic drug resistance proteins (PDR1-15), ABC superfamily [Q] |
|  | >LELT_05276 \| transcript encoding Lodderomyces elongisporus hypothetical protein (903 nt) | G19 | 0.93 | Cell division protein FtsJ [D] |
| *M. grisea* | >MGT_00609 \| transcript encoding Magnaporthe grisea hypothetical protein (3135 nt) | T15 | 0.06 |  |
|  | >MGT_01897 \| transcript encoding Magnaporthe grisea predicted protein (255 nt) | G18 | 0.54 |  |
|  | >MGT_02764 \| transcript encoding Magnaporthe grisea predicted protein (162 nt) | G15 | 0.73 |  |
|  | >MGT_03680 \| transcript encoding Magnaporthe grisea predicted protein (2229 nt) | T15 | 0.04 |  |
|  | >MGT_05257 \| transcript encoding Magnaporthe grisea predicted protein (666 nt) | T15 | 0.07 |  |
|  | >MGT_06860 \| transcript encoding Magnaporthe grisea predicted protein (336 nt) | G19 | 0.7 |  |
|  | >MGT_06950 \| transcript encoding Magnaporthe grisea predicted protein (168 nt) | G18 | 0.24 |  |
|  | >MGT_08718 \| transcript encoding Magnaporthe grisea hypothetical protein (1539 nt) | G16 | 0.68 | Acetylornithine aminotransferase [E] |
|  | >MGT_12729 \| transcript encoding Magnaporthe grisea predicted protein (372 nt) | G17 | 0.61 |  |
| *N. fischeri* | >null \| transcript encoding Neosartorya fischeri null (783 nt) 52701 | C16 | 0.86 |  |
| *N. crassa* | >NCUT_00950 \| transcript encoding Neurospora crassa histone acetyltransferase GCN5 (1269 nt) | C27 | 0.04 | Histone acetyltransferase SAGA/ADA, catalytic subunit PCAF/GCN5 and related proteins [BK] |
|  | >NCUT_04676 \| transcript encoding Neurospora crassa predicted protein (360 nt) | G18 | 0.92 |  |
|  | >NCUT_07413 \| transcript encoding Neurospora crassa predicted protein (435 nt) | G26 | 0.07 |  |
|  | >NCUT_09476 \| transcript encoding Neurospora crassa conserved hypothetical protein (318 nt) | C22 | 0.75 | Small nuclear ribonucleoprotein F [A] |
| *P. brasiliensis pb03* | >PABT_03968 \| transcript encoding Paracoccidioides brasiliensis Pb03 predicted protein (930 nt) | G18 | 0.03 |  |
|  | >PABT_06070 \| transcript encoding Paracoccidioides brasiliensis Pb03 sporulation protein RMD1 (1749 nt) | G19 | 0.93 | Uncharacterized conserved protein [S] |
|  | >PABT_06018 \| transcript encoding Paracoccidioides brasiliensis Pb03 predicted protein (513 nt) | A37 | 0.63 |  |
|  | >PABT_07580 \| transcript encoding Paracoccidioides brasiliensis Pb03 conserved hypothetical protein (1263 nt) | C17 | 0.50 |  |
|  | >PABT_00782 \| transcript encoding Paracoccidioides brasiliensis Pb03 predicted protein (276 nt) | A19 | 0.14 |  |
|  | >PABT_00782 \| transcript encoding Paracoccidioides brasiliensis Pb03 predicted protein (276 nt) | A17 | 0.44 |  |
| *P. graminis f. sp. tritici* | >PGTT_18133 \| transcript encoding Puccinia graminis f. sp. tritici predicted protein (939 nt) | A15 | 0.74 |  |
|  | >PGTT_14663 \| transcript encoding Puccinia graminis f. sp. tritici hypothetical protein (2466 nt) | T16 | 0.98 |  |
|  | >PGTT_09195 \| transcript encoding Puccinia graminis f. sp. tritici conserved hypothetical protein (1011 nt) | T16 | 0.60 | Uncharacterized conserved protein [S] |
|  | >PGTT_10592 \| transcript encoding Puccinia graminis f. sp. tritici predicted protein (1131 nt) | A15 | 0.81 |  |
|  | >PGTT_12921 \| transcript encoding Puccinia graminis f. sp. tritici predicted protein (429 nt) | G18 | 0.11 |  |
|  | >PGTT_10636 \| transcript encoding Puccinia graminis f. sp. tritici predicted protein (300 nt) | T18 | 0.10 | FOG: Cadherin repeats [S] |
| *R. oryzae* | >RO3T_01599 \| transcript encoding Rhizopus oryzae hypothetical protein (201 nt) | A33 | 0.27 |  |
|  | >RO3T_01657 \| transcript encoding Rhizopus oryzae hypothetical protein (489 nt) | T21 | 0.09 |  |
|  | >RO3T_01687 \| transcript encoding Rhizopus oryzae hypothetical protein (2115 nt) | A17 | 0.99 | Protein involved in vacuole import and degradation [U] |
|  | >RO3T_03375 \| transcript encoding Rhizopus oryzae hypothetical protein (1278 nt) | T16 | 0.98 | Signal transduction serine/threonine kinase with PAS/PAC sensor domain [T] |
|  | >RO3T_03847 \| transcript encoding Rhizopus oryzae hypothetical protein (357 nt) | T16 | 0.08 | ATP-dependent RNA helicase [A] |
|  | >RO3T_05259 \| transcript encoding Rhizopus oryzae hypothetical protein (951 nt) | T16 | 0.02 |  |
|  | >RO3T_06859 \| transcript encoding Rhizopus oryzae predicted protein (252 nt) | T17 | 0.17 |  |
|  | >RO3T_09677 \| transcript encoding Rhizopus oryzae hypothetical protein (285 nt) | A24 | 0.91 | U1 small nuclear ribonucleoprotein (RRM superfamily) [A] |
|  | >RO3T_10115 \| transcript encoding Rhizopus oryzae hypothetical protein (567 nt) | T18 | 0.79 | Uncharacterized conserved protein [S] |
|  | >RO3T_12442 \| transcript encoding Rhizopus oryzae predicted protein (780 nt) | A23 | 0.51 |  |
|  | >RO3T_16247 \| transcript encoding Rhizopus oryzae hypothetical protein (6249 nt) | T25 | 0.13 | Glutamate synthase [E] |
| *S. cerevisiae rm11-1a* | >SCRT_03731 \| transcript encoding Saccharomyces cerevisiae RM11-1a conserved hypothetical protein (330 nt) 95939 | T16 | 0.17 |  |
|  | >SCRT_03248 \| transcript encoding Saccharomyces cerevisiae RM11-1a predicted protein (180 nt) | A26 | 0.19 |  |
| *S. octosporus yfs286* | >SOCG_01255T0 \| transcript encoding Schizosaccharomyces octosporus yFS286 ribonuclease P 21 subunit (345 nt) | A16 | 0.93 | RNase P subunit that is not also a subunit of RNase MRP, involved in pre-tRNA processing [A] |
| *S. pombe 972h* | >SPAPB15E9_02c.1 \| transcript encoding Schizosaccharomyces pombe 972h- dubious (567 nt) | T25 | 0.43 |  |
|  | >SPAC17A2_10c.1 \| transcript encoding Schizosaccharomyces pombe 972h- sequence orphan (693 nt) | T16 | 0.68 |  |
| *S. sclerotiorum* | >SS1T_05416 \| transcript encoding Sclerotinia sclerotiorum predicted protein (165 nt) | G15 | 0.33 |  |
|  | >SS1T_09507 \| transcript encoding Sclerotinia sclerotiorum predicted protein (3528 nt) | G15 | 0.92 | Myosin class II heavy chain [Z] |
|  | >SS1T_11730 \| transcript encoding Sclerotinia sclerotiorum predicted protein (654 nt) | G16 | 0.97 |  |
|  | >SS1T_01990 \| transcript encoding Sclerotinia sclerotiorum predicted protein (318 nt) | G20 | 0.07 |  |
|  | >SS1T_12372 \| transcript encoding Sclerotinia sclerotiorum hypothetical protein (651 nt) | C20 | 0.86 | a) Protein kinase PCTAIRE and related kinases [R]  b) Mitogen-activated protein kinase [T]  c) Nemo-like MAPK-related serine/threonine protein kinase [T]  d) Jun-N-terminal kinase (JNK) [T]  e) LAMMER dual specificity kinases [T] |
| *S. nodorum* | >SNOT_08033 \| transcript encoding Stagonospora nodorum predicted protein (528 nt) | C18 | 0.16 |  |
| *U. reesii* | >URET_07408 \| transcript encoding Uncinocarpus reesii predicted protein (603 nt) | A16 | 0.88 |  |
|  | >URET_07473 \| transcript encoding Uncinocarpus reesii predicted protein (246 nt) | A16 | 0.24 |  |
| *U. maydis* | >null \| transcript encoding Ustilago maydis hypothetical protein (891 nt) | C17 | 0.75 |  |
|  | >null \| transcript encoding Ustilago maydis hypothetical protein (2943 nt) | T25 | 0.01 | Phosphatidylserine-specific receptor PtdSerR, contains JmjC domain [BT] |
| *V. albo-atrum vams.102* | >VDBG_07075T0 \| transcript encoding Verticillium albo-atrum VaMs.102 ATP-dependent RNA helicase DBP3 (1716 nt) | A25 | 0.03 | ATP-dependent RNA helicase [A] |
| *V. dahliae vdls.17* | >VDAG_01742T0 \| transcript encoding Verticillium dahliae VdLs.17 hypothetical protein (453 nt) | A22 | 0.44 |  |
|  | >VDAG_02915T0 \| transcript encoding Verticillium dahliae VdLs.17 conserved hypothetical protein (813 nt) | T16 | 0.91 |  |
|  | >VDAG_03108T0 \| transcript encoding Verticillium dahliae VdLs.17 conserved hypothetical protein (966 nt) | C23 | 0.7 | Uncharacterized conserved protein [S] |
|  | >VDAG_05416T0 \| transcript encoding Verticillium dahliae VdLs.17 guanyl-specific ribonuclease F1 (369 nt) | C23 | 0.85 |  |
|  | >VDAG_08703T0 \| transcript encoding Verticillium dahliae VdLs.17 alpha-1,2 mannosyltransferase KTR1 (729 nt) | G18 | 0.85 | Glycolipid 2-alpha-mannosyltransferase (alpha-1,2-mannosyltransferase) [G] |
|  | >VDAG_08991T0 \| transcript encoding Verticillium dahliae VdLs.17 proteasome component PUP2 (321 nt) | G15 | 0.34 | 20S proteasome, regulatory subunit alpha type PSMA5/PUP2 [O] |
|  | >VDAG_09974T0 \| transcript encoding Verticillium dahliae VdLs.17 CaaX farnesyltransferase alpha subunit (1725 nt) | C18 | 0.1 | Protein farnesyltransferase, alpha subunit/protein geranylgeranyltransferase type I, alpha subunit [O] |

*) Transcripts without a unique gene classifier (such as in *A. clavatus*, *A. fumigatus*, *A. oryzae* and *M. grisea*) are combined with their line number in their respective fasta file in order to process unique entries in the analyses
