# Supplementary material for: Mononucleotide repeats are asymmetrically distributed in fungal genes
Source: BMC Genomics. 2008 Dec 11;9:596. doi: 10.1186/1471-2164-9-596 (PMC2621210; doi:10.1186/1471-2164-9-596)
Supplement: Additional File 3 — Repertoire sizes of genes with different repeat lengths. Gene repertoire sizes of the fungi, and the counts and fractions of genes that contain repeats of lengths five until greater than nine. [file 1471-2164-9-596-S3.docx]

**Additional file 3**.

File format: doc file

Title: Repertoire sizes of genes with different repeat lengths

Description: Gene repertoire sizes of the fungi, and the counts and fractions of genes that contain repeats of lengths five until greater than nine.

|  | **Gene counts per genome containing repeats of said length** | | | | | | | **Genome fractions containing repeats of said length (%)** | | | | | | |
| --- | --- | --- | --- | --- | --- | --- | --- | --- | --- | --- | --- | --- | --- | --- |
| **Species** | **All CDS** | **5** | **6** | **7** | **8** | **9** | **>9** | **All CDS** | **5** | **6** | **7** | **8** | **9** | **>9** |
| Ac | 9110 | 7965 | 3644 | 820 | 129 | 40 | 21 | 100 | 87.43 | 40.00 | 9.00 | 1.42 | 0.44 | 0.23 |
| Afl | 12434 | 10634 | 4521 | 891 | 138 | 30 | 10 | 100 | 85.52 | 36.36 | 7.17 | 1.11 | 0.24 | 0.08 |
| Afu | 9884 | 8586 | 3865 | 986 | 219 | 98 | 62 | 100 | 86.87 | 39.10 | 9.98 | 2.22 | 0.99 | 0.63 |
| Anid | 10474 | 9137 | 4137 | 971 | 186 | 39 | 11 | 100 | 87.24 | 39.50 | 9.27 | 1.78 | 0.37 | 0.11 |
| Anig | 6329 | 5716 | 2532 | 523 | 106 | 56 | 38 | 100 | 90.31 | 40.01 | 8.26 | 1.67 | 0.88 | 0.60 |
| Ao | 12063 | 10402 | 4393 | 901 | 176 | 50 | 23 | 100 | 86.23 | 36.42 | 7.47 | 1.46 | 0.41 | 0.19 |
| At | 10400 | 9279 | 4023 | 617 | 73 | 21 | 11 | 100 | 89.22 | 38.68 | 5.93 | 0.70 | 0.20 | 0.11 |
| Bd | 8779 | 8169 | 6050 | 3106 | 1104 | 212 | 30 | 100 | 93.05 | 68.91 | 35.38 | 12.58 | 2.41 | 0.34 |
| Bc | 15512 | 12409 | 6004 | 1634 | 305 | 95 | 46 | 100 | 80.00 | 38.71 | 10.53 | 1.97 | 0.61 | 0.30 |
| Ca_sc | 5916 | 5762 | 4615 | 2255 | 688 | 250 | 137 | 100 | 97.40 | 78.01 | 38.12 | 11.63 | 4.23 | 2.32 |
| Ca_wo | 5851 | 5690 | 4543 | 2178 | 630 | 203 | 94 | 100 | 97.25 | 77.64 | 37.22 | 10.77 | 3.47 | 1.61 |
| Cgu | 5897 | 5622 | 3845 | 1458 | 349 | 64 | 13 | 100 | 95.34 | 65.20 | 24.72 | 5.92 | 1.09 | 0.22 |
| Cl | 5891 | 5437 | 3379 | 1250 | 355 | 142 | 70 | 100 | 92.29 | 57.36 | 21.22 | 6.03 | 2.41 | 1.19 |
| Cp | 5687 | 5468 | 3730 | 1494 | 480 | 203 | 114 | 100 | 96.15 | 65.59 | 26.27 | 8.44 | 3.57 | 2.00 |
| Ct | 6216 | 5981 | 4369 | 1680 | 499 | 257 | 187 | 100 | 96.22 | 70.29 | 27.03 | 8.03 | 4.13 | 3.01 |
| Cgl | 10987 | 9932 | 6092 | 2619 | 758 | 198 | 58 | 100 | 90.40 | 55.45 | 23.84 | 6.90 | 1.80 | 0.53 |
| Cih | 10450 | 8828 | 4028 | 1092 | 261 | 63 | 30 | 100 | 84.48 | 38.55 | 10.45 | 2.50 | 0.60 | 0.29 |
| Ci_2394 | 10368 | 8946 | 4363 | 1226 | 302 | 76 | 38 | 100 | 86.28 | 42.08 | 11.82 | 2.91 | 0.73 | 0.37 |
| Ci_3703 | 10379 | 8772 | 4027 | 1114 | 278 | 82 | 41 | 100 | 84.52 | 38.80 | 10.73 | 2.68 | 0.79 | 0.40 |
| Ci_rs | 10609 | 9199 | 4528 | 1285 | 311 | 85 | 41 | 100 | 86.71 | 42.68 | 12.11 | 2.93 | 0.80 | 0.39 |
| Cp_3488 | 9932 | 8589 | 4215 | 1163 | 271 | 93 | 38 | 100 | 86.48 | 42.44 | 11.71 | 2.73 | 0.94 | 0.38 |
| Cp_silvereira | 10070 | 8668 | 4197 | 1132 | 269 | 80 | 37 | 100 | 86.08 | 41.68 | 11.24 | 2.67 | 0.79 | 0.37 |
| Cc | 13523 | 11202 | 4209 | 638 | 105 | 31 | 10 | 100 | 82.84 | 31.12 | 4.72 | 0.78 | 0.23 | 0.07 |
| Cn | 7077 | 6399 | 3686 | 1148 | 254 | 60 | 19 | 100 | 90.42 | 52.08 | 16.22 | 3.59 | 0.85 | 0.27 |
| Dh | 6101 | 5682 | 3926 | 1702 | 483 | 113 | 35 | 100 | 93.13 | 64.35 | 27.90 | 7.92 | 1.85 | 0.57 |
| Fg | 13220 | 10695 | 3757 | 709 | 113 | 33 | 15 | 100 | 80.90 | 28.42 | 5.36 | 0.85 | 0.25 | 0.11 |
| Fo | 17202 | 13218 | 4440 | 905 | 114 | 32 | 16 | 100 | 76.84 | 25.81 | 5.26 | 0.66 | 0.19 | 0.09 |
| Fv | 14002 | 10849 | 3635 | 684 | 105 | 17 | 7 | 100 | 77.48 | 25.96 | 4.89 | 0.75 | 0.12 | 0.05 |
| Hc | 9164 | 8146 | 4686 | 1601 | 403 | 114 | 44 | 100 | 88.89 | 51.13 | 17.47 | 4.40 | 1.24 | 0.48 |
| Le | 5739 | 5457 | 3903 | 1820 | 605 | 207 | 113 | 100 | 95.09 | 68.01 | 31.71 | 10.54 | 3.61 | 1.97 |
| Mg | 12564 | 10464 | 4038 | 714 | 199 | 48 | 29 | 100 | 83.29 | 32.14 | 5.68 | 1.58 | 0.38 | 0.23 |
| Nf | 10383 | 9025 | 3648 | 626 | 81 | 13 | 3 | 100 | 86.92 | 35.13 | 6.03 | 0.78 | 0.13 | 0.03 |
| Nc | 9795 | 8428 | 3975 | 927 | 107 | 30 | 12 | 100 | 86.04 | 40.58 | 9.46 | 1.09 | 0.31 | 0.12 |
| Pb | 9235 | 8123 | 4494 | 1564 | 413 | 116 | 45 | 100 | 87.96 | 48.66 | 16.94 | 4.47 | 1.26 | 0.49 |
| Pg | 20462 | 18441 | 11169 | 4040 | 1243 | 476 | 200 | 100 | 90.12 | 54.58 | 19.74 | 6.07 | 2.33 | 0.98 |
| Pt | 12092 | 9726 | 3889 | 748 | 93 | 15 | 5 | 100 | 80.43 | 32.16 | 6.19 | 0.77 | 0.12 | 0.04 |
| Ro | 17074 | 15198 | 9799 | 3957 | 1244 | 392 | 128 | 100 | 89.01 | 57.39 | 23.18 | 7.29 | 2.30 | 0.75 |
| Sce | 5272 | 5009 | 3904 | 2272 | 985 | 341 | 111 | 100 | 95.01 | 74.05 | 43.10 | 18.68 | 6.47 | 2.11 |
| Sj | 5122 | 4603 | 3116 | 1280 | 341 | 71 | 13 | 100 | 89.87 | 60.84 | 24.99 | 6.66 | 1.39 | 0.25 |
| So | 4906 | 4641 | 3538 | 1824 | 628 | 160 | 31 | 100 | 94.60 | 72.12 | 37.18 | 12.80 | 3.26 | 0.63 |
| Sp | 4991 | 4809 | 3878 | 2222 | 886 | 279 | 85 | 100 | 96.35 | 77.70 | 44.52 | 17.75 | 5.59 | 1.70 |
| Scl | 13704 | 11235 | 5603 | 1536 | 344 | 86 | 38 | 100 | 81.98 | 40.89 | 11.21 | 2.51 | 0.63 | 0.28 |
| Sn | 15949 | 11449 | 3702 | 706 | 102 | 21 | 9 | 100 | 71.79 | 23.21 | 4.43 | 0.64 | 0.13 | 0.06 |
| Ur | 7777 | 6973 | 3575 | 922 | 169 | 32 | 17 | 100 | 89.66 | 45.97 | 11.86 | 2.17 | 0.41 | 0.22 |
| Um | 6517 | 5335 | 2068 | 449 | 61 | 19 | 10 | 100 | 81.86 | 31.73 | 6.89 | 0.94 | 0.29 | 0.15 |
| Va | 10098 | 8296 | 3374 | 940 | 221 | 85 | 51 | 100 | 82.15 | 33.41 | 9.31 | 2.19 | 0.84 | 0.51 |
| Vd | 10453 | 8827 | 3627 | 938 | 168 | 63 | 36 | 100 | 84.44 | 34.70 | 8.97 | 1.61 | 0.60 | 0.34 |
